# Nanovesicles With Mechanically Induced Adjuvanticity for Robust Melanoma Vaccination Toward Tumor‐Associated Macrophages

**DOI:** 10.1002/advs.76773

**Published:** 2026-07-27

**Authors:** Bangyue Luo, Liyan Qiu

**Affiliations:** ^1^ Department of Polymer Science and Engineering Ministry of Educational (MOE) Key Laboratory of Macromolecular Synthesis and Functionalization Zhejiang University Hangzhou China

**Keywords:** immunosuppression reversal, mechanosensitive signaling pathway, melanoma vaccine, nanovesicle adjuvants, tumor‐associated macrophages

## Abstract

Tumor‐associated macrophages (TAMs) are abundant in tumor microenvironment (TME) but fail to act as vaccine targets due to their inferior immune response. Here, a robust strategy was presented to develop potent cancer vaccines toward TAMs via a multi‐functional adjuvant, breaking through the conventional vaccine design centered on dendritic cells (DCs). To address this issue, rigidity‐tunable nanovesicles (P‐P_m_) were engineered by surface decoration of Chol‐PItEG_m_ with precise regulation of chain lengths. The P‐P_m_ nanovesicle exerted mechanically induced adjuvanticity through not only enhancing endocytosis in a rigidity‐dependent manner to facilitate antigen delivery and processing in TAMs, but also activating the mechanosensitive Piezo1/YAP/TAZ signaling pathway to promote M2‐like TAMs reprogramming. Furthermore, the rigid P‐P_m_ nanovesicle could efficiently co‐encapsulate antigenic gp100 peptide and toll‐like receptor agonist resiquimod (R848) to form an anti‐melanoma vaccine P@Rg‐P_m_. After intravenously injected, CD8^+^ T cell infiltration was increased while immune‐suppressive cell level of myeloid derived suppressor cells (MDSCs) and regulatory T cells (Tregs) were decreased along with elevated levels of proinflammatory factors including IL‐1β, IFN‐γ, and IL‐12, demonstrating the effective reversal of immunosuppressive TME. Consequently, P@Rg‐P_m_ demonstrates significant antitumor efficacy against B16‐F10 melanoma in both therapeutic and prophylactic models without any assistance of other therapies.

## Graphical abstract

Rigidity‐tunable nanovesicle (P‐P_m_) with mechanically induced adjuvanticity toward tumor‐associated macrophages (TAMs) was designed to engineer R848/gp100 antigen ‐loaded nanovaccine (P@Rg‐P_m_), which achieved outstanding anti‐tumor outcomes in both therapeutic and preventive model of B16‐F10 melanoma.

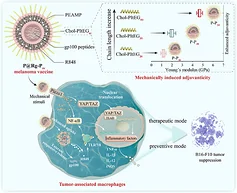

## 1 Introduction

As a promising immunotherapeutic strategy, cancer vaccines have been extensively studied in recent years, aiming to activate the autologous immune system and elicit immune responses against tumors [1, 2]. In the general immunity process, antigen presenting cells (APCs), mostly dendritic cells (DCs), internalize tumor antigens and display major histocompatibility complex (MHC)‐peptide complex on their surface, which induces antigen‐specific T cells to recognize and kill tumor cells. Nevertheless, the immunosuppressive tumor microenvironment (TME) with impaired immune cell function and fostered resistance severely compromises the therapeutic efficacy of cancer vaccine in monotherapy model unless with assistance of other therapies [3]. Therefore, more advances are urgently needed for cancer vaccines to achieve significant results.

Apart from DCs, macrophages are another kind of professional APCs, but they are almost ignored in the field of cancer vaccine [4]. Tumor‐associated macrophages (TAMs), originated from circulating monocytes and tissue resident macrophages, are special macrophages educated by TME. It was reported that TAMs represent up to 50% of immune cells in solid tumors, but they play a predominant role in immune resistance [5]. Different from DCs, the inherent plasticity and heterogeneity of TAMs allow them to display M1‐ or M2‐like phenotype. M1‐like TAMs with relatively weak lysosome acidification and proteolysis greatly potentiates their antigen cross‐presentation abilities and effectively stimulate CD8^+^ T cell function for cancer immunotherapy [6]. Conversely, M2‐like TAMs suffer from antigen presentation deficiency, which is highly correlated with immune resistance [7]. Unfortunately, various signals in tumors activate intracellular transcriptional cross talk in TAMs, leading to the augment of M2 macrophage population [8, 9]. Clinical evidence reveals that M2‐polarized TAMs frequently constitute the majority (up to 70–80%) of tumor‐infiltrating macrophages across various solid malignancies [10, 11]. In addition, M1‐like TAMs secret proinflammatory cytokines such as TNF‐α, IL‐1β, IL‐12 and IL‐23 to promote T cell responses, while M2‐like TAMs could promote tumor progression via an array of effector molecules including specific cell surface receptors, cytokines, chemokines, and enzymes, which in turn aggravate immune resistance [12]. Therefore, enhancing the antigen‐presenting capacity of TAMs is a crucial point for developing an excellent cancer vaccine against solid tumors. Currently, a number of researches have been performed on the issue how to reverse the immunosuppressive microenvironment by reprogramming M2‐like TAMs to M1‐like TAMs [13, 14]. However, the recovery and utilization of antigen presenting function of TAMs for cancer vaccines is scarcely explored.

Designing and optimizing adjuvants is essential to exploit TAMs as antigen‐presenting cells in cancer vaccines. Adjuvants are indispensable components for cancer vaccines, serving as critical immune potentiators to overcome the weak immunogenicity and enhance cancer immunotherapy [15]. By targeting innate immune cells and activating pattern recognition receptor signaling pathways, adjuvants orchestrate and amplify antigen‐specific adaptive immune responses [16]. Current adjuvant strategies predominantly employ Toll‐like receptor (TLR) agonists (e.g., resiquimod (R848)) and STING agonists (e.g., cGAMP), yet these approaches confront some troubles including systemic toxicity of free molecular adjuvants (manifested as inflammatory storms) and low delivery efficiency [17, 18]. Also, some particulate adjuvants, such as cationic liposomes, oil‐in‐water emulsions, aluminum salt particles, and mesoporous silica nanoparticles, have been explored in cancer vaccines to enhance antigen delivery to DCs and stimulate robust immune response [19, 20, 21]. These studies provided some valuable references for adjuvant design, but more issues such as TAMs repolarization are to be taken into account for cancer vaccine.

Here, we engineered P@Rg‐P_m_ nanovesicles with mechanically induced adjuvanticity for a melanoma vaccine that integrates: (1) tunable rigidity to activate mechanotransduction pathways, (2) mechano‐related transportation into TAMs, (3) mechanosensitive TAMs reprogramming, and (4) immunosuppressive TME reversal, collectively achieving potent adjuvanticity to enhance immunotherapy against melanoma. As illustrated in Scheme 1, the Nanovesicles were self‐assembled by amphiphilic poly[(methoxy‐poly(ethylene glycol))(2‐aminoethyl methacrylate) phosphazene]s (PEAMP), along with the decoration of cholesterol‐poly(4‐isocyanobenzoic acid tetraethylene glycol monomethyl ether ester)_m_ (Chol‐PItEG_m_) on the surface. The Chol‐PItEG_m_ chain presented a helical conformation, which displayed specific rigidity owing to intermolecular forces and spatial hindrance. By modulating the chain length of Chol‐PItEG_m_, we achieved precise control over nanovesicles rigidity, resulting in Young's moduli ranging from 0.69 ± 0.05 to 4.65 ± 0.17 GPa. These rigidity‐tunable Nanovesicles generated strong interactions with TAMs and reprogrammed immunosuppressive M2 phenotypes toward immunostimulatory M1 states in a stiffness‐dependent manner. This reprogramming was mediated by the mechanosensitive Piezo1 ion channel and the mechanotransduction‐associated transcriptional coactivator YAP/TAZ. Furthermore, the Nanovesicles with a core–shell structure owned excellent drug loading capability to co‐encapsulating hydrophobic R848 and water‐soluble antigenic peptides gp100 to form P@Rg‐P_m_. Herein, gp100 peptide is a classic tumor‐associated antigen expressed by melanoma B16‐F10 cells. R848, a dual TLR7 and TLR8 synthetic agonist, activates immune cells as a biochemical stimulus. Markedly, P@Rg‐P_m_ was designed to be sensitive to the abundant esterase in TAMs, thereby triggering the intracellular R848 release for TLR7/8 stimulation, further inducing M1 polarization at the biochemical level. Benefiting from Nanovesicles with mechanically induced adjuvanticity and biochemical signaling activation, P@Rg‐P_m_ demonstrated pronounced tumor inhibition in both prophylactic and therapeutic murine melanoma models, offering a comprehensive and efficient strategy to enhance immunotherapy outcomes in melanoma treatment.

**SCHEME 1:**
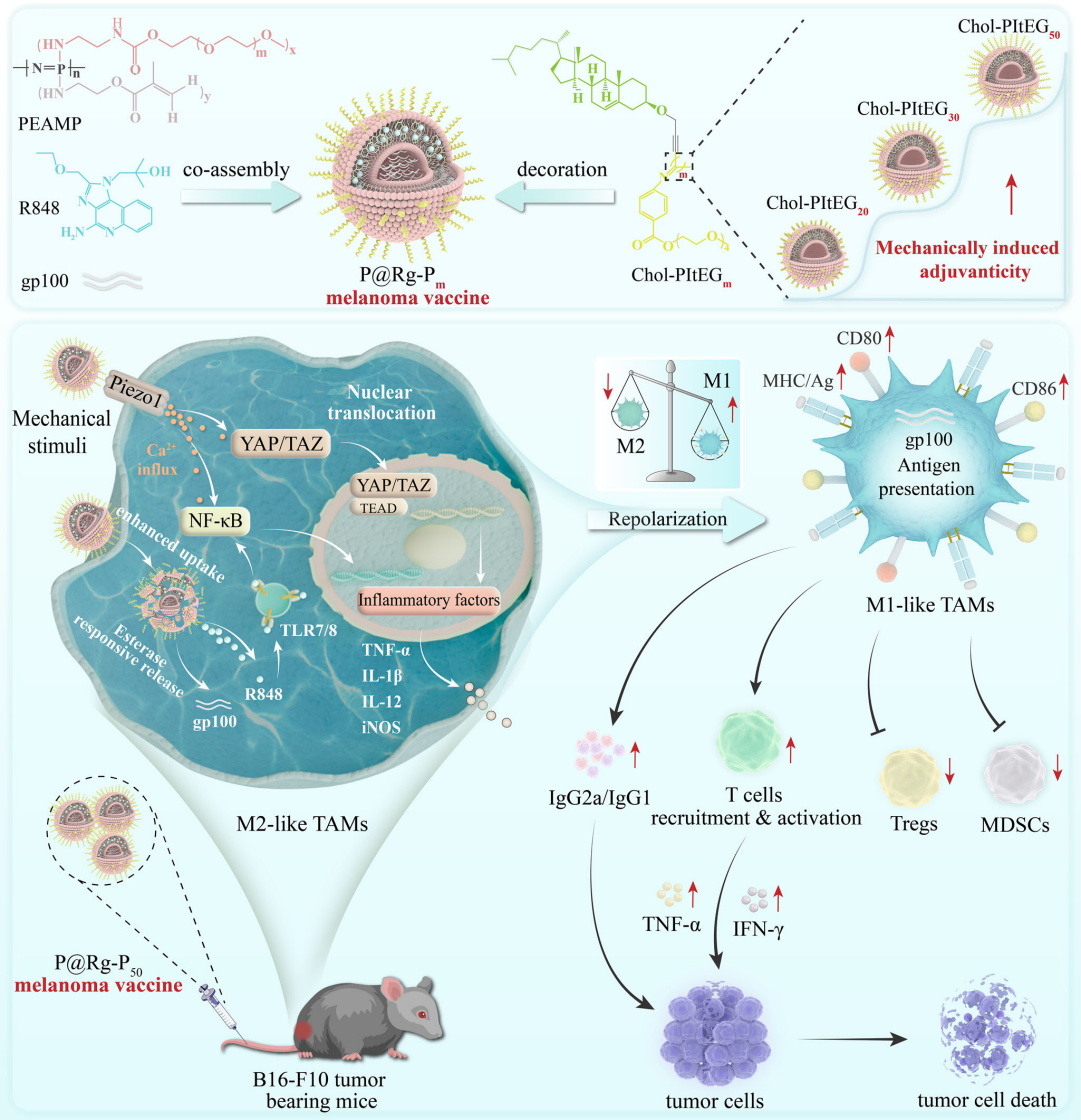
Schematic illustration of melanoma vaccine design for melanoma immunotherapy by P@Rg‐P_m_ Nanovesicles with mechanically induced adjuvanticity.

## 2 Results and Discussion

### 2.1 Preparation and Characterization of P@Rg‐P_m_

For constructing P@Rg‐P_m_ nanovesicles, the amphiphilic polyphosphazene PEAMP was first synthesized through the sequential substitution reactions of mPEG_2000_‐NH_2_/AEMA with the chlorine atoms on the poly(dichlorophosphazene) backbone (Figures S1 and S3) [22]. Successful synthesis of PEAMP was verified by ^1^H nuclear magnetic resonance (^1^H‐NMR) and fourier transform infrared (FT‐IR) analysis (Figures S2, S4 and S5). Subsequently, dynamic light scattering (DLS) and transmission electron microscopy (TEM) images show that PEAMP could self‐assemble in aqueous solution, characterized by a typical nanovesicles morphology with a hydrophilic cavity encased by a hydrophobic membrane (Figure 1A). Further supporting evidence came from confocal fluorescence microscopy (Figure 1B) that hydrophobic Nile red was localized at the outer ring (membrane) and water‐soluble gp100‐FITC concentrated in the core (cavity), conclusively demonstrating the vesicular architecture. This vesicular architecture enables dual‐payload co‐delivery and spatial segregation, which is a prerequisite to melanoma vaccination design. By the solvent exchange method, hydrophobic R848 and hydrophilic gp100 peptides were simultaneously loaded into the membrane and cavity of PEAMP nanovesicles, respectively (denoted as P@Rg) (Figure 1A). The loading capacity (LC) was 4.97% ± 0.23% for R848 and 3.58% ± 0.51% for gp100 peptides with the corresponding encapsulation efficiency (EE) of 54.44% ± 2.90% and 78.39% ± 11.74%.

**FIGURE 1:**
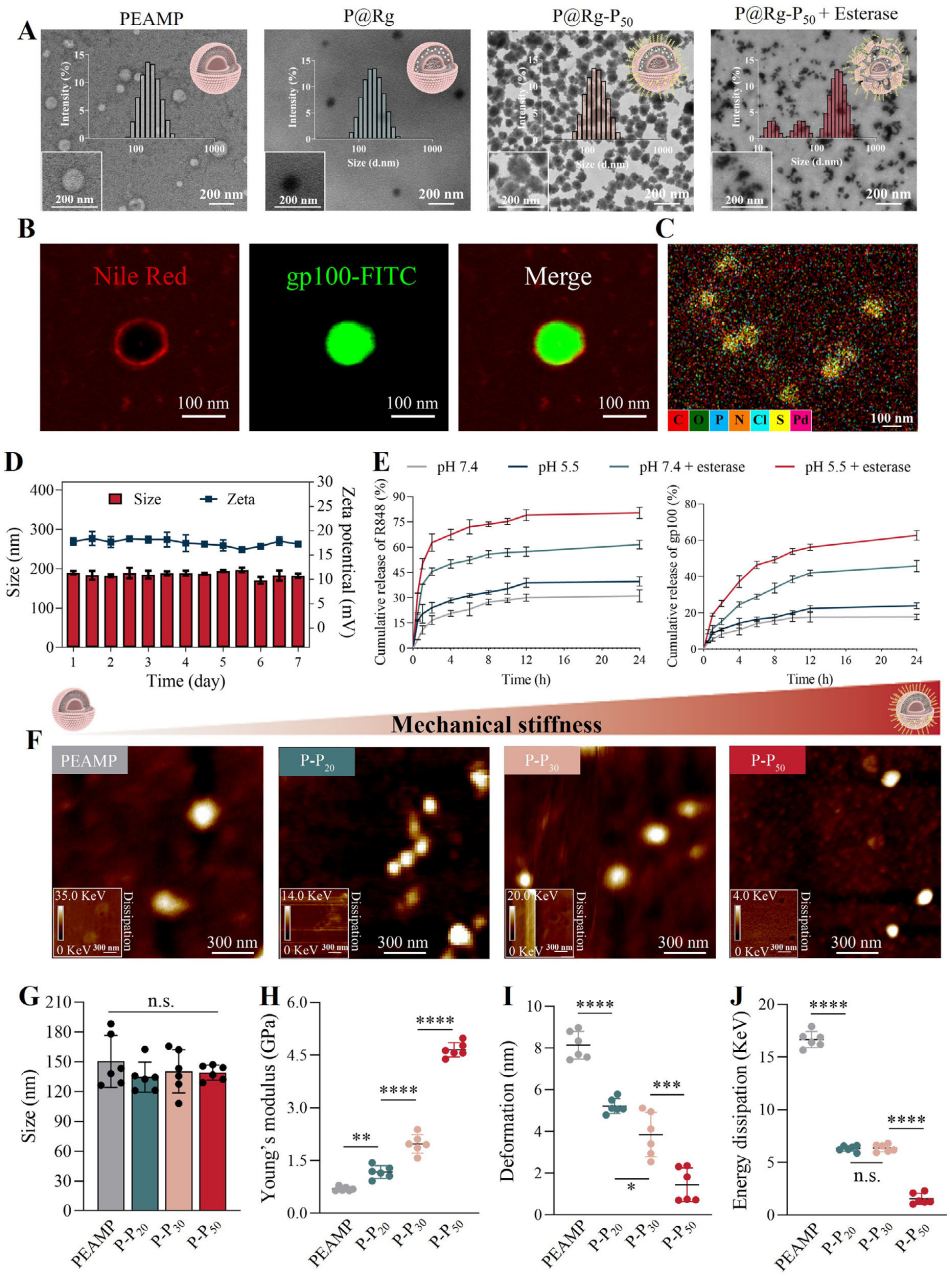
Preparation and characterization of P@Rg‐P_m_. (A) Size distribution and corresponding TEM images of PEAMP, P@Rg, P@Rg‐P_50_, and P@Rg‐P_50_ + esterase (1500 ng mL^−1^), scale bar: 200 nm. (B) Confocal fluorescence images of PEAMP nanovesicles loaded with Nile Red and gp100‐FITC, scale bar: 100 nm. (C) Elemental analysis of P@Rg‐P_50_ by EDS, scale bar: 100 nm. (D) Size and zeta potential stability of P@Rg‐P_50_ at 4°C for 7 days. (E) Release profile of R848 or gp100 peptides in the presence or absence of esterase (1500 ng mL^−1^) at pH 5.5 or 7.4 for 24 h. (F) Topography images and energy dissipation maps of PEAMP, P‐P_20_, P‐P_30_, and P‐P_50_ from AFM scanning, scale bar: 300 nm. (G–J) The size distribution, Young's modulus, deformation, and energy dissipation analysis of PEAMP, P‐P_20_, P‐P_30_, and P‐P_50_ from AFM scanning (*n* = 6). Data are presented as mean ± s.d. Statistical significance was determined by one‐way ANOVA with Tukey's multiple‐comparisons test. ^*^ *p* < 0.05, ^**^ *p* < 0.01, ^***^ *p* < 0.001, and ^****^ *p* < 0.0001; n.s., not significant.

For endowing P@Rg nanovesicles with enhanced structural rigidness, stiff helical chains Chol‐PItEG_m_ were designed for surface decoration. Chol‐PItEG_m_ was synthesized by Chol‐Pd(II) complex initiated living polymerization of 4‐isocyanobenzoic acid tetraethylene glycol monomethyl ether ester (ItEG) (Figures S6, S10, and S12). The rigidity of Chol‐PItEG_m_ stems from its unique polyisocyanide backbone. The distinctive π‐conjugated system of ItEG, characterized by C = N double bonds linking the monomers, can induce a twist in the polyisocyanide backbone during polymerization, forming a helical conformation [23, 24, 25]. This helical arrangement presents fascinating rigidity, pivotal for maintaining structural stability and enhancing mechanical properties [26, 27, 28]. Moreover, the Chol‐Pd(II) complex acts as an initiator, ensuring their controlled chain length and high stereoregularity, which maintains the helical morphology of the Chol‐PItEG_m_ chain [23]. Successful synthesis of the ItEG monomer and Chol‐Pd(II) initiator was confirmed by ^1^H‐NMR (Figures S7–S9 and S11). By adjusting the initiator‐to‐monomer ratio, Chol‐PItEG_m_ with different chain length were synthesized, with polymerization degrees (DP) of 24, 32, and 55 (denoted as Chol‐PItEG_20_, Chol‐PItEG_30_, and Chol‐PItEG_50_), respectively (Figures S12 and Tables S1 and S2). FT‐IR spectra demonstrated that Chol‐PItEG_m_ was successfully polymerized, evidenced by the peak at 1600 cm^−1^ ascribed to the C═N vibration of the polyisocyanide backbone (Figure S13). ^1^H‐NMR signals also supports the structural assignment of Chol‐PItEG_m_ (Figure S14). Moreover, gel permeation chromatography (GPC) results confirmed that the chain growth of Chol‐PItEG_m_ was polymerized in a controlled manner, with a desire DP and a narrow polydispersity index (PDI) (Figure S15 and Table S2). Contact angle analysis reveals comparable hydrophobicity across Chol‐PItEG_m_ variants with differing chain lengths, consistently maintaining hydrophilic dominance (Figure S16).

The pre‐formed P@Rg nanovesicles were then surface‐modified by Chol‐PItEG_m_ via hydrogen bonding and hydrophobic interaction between the cholesterol moiety of Chol‐PItEG_m_ and the AEMA groups within the hydrophobic membrane of the PEAMP nanovesicles. DLS analysis suggests that the resulting P@Rg‐P_m_ with different chain length (P@Rg‐P_20_, P@Rg‐P_30_, and P@Rg‐P_50_) show no significant differences in size distribution and zeta potential (Table S3). All variants shared comparable physicochemical properties, allowing the subsequent analyses to focus on rigidity‐dependent immunological effects. TEM revealed that surface decoration with the Chol‐PItEG_50_ chains dramatically altered the morphology of the nanovesicles. P@Rg‐P_50_ exhibited distinctive nanoscale protrusions and an irregular surface (Figure 1A), a stark contrast to the smooth surface of the unmodified P@Rg nanovesicles. SEM images further corroborated this observation, clearly showing enhanced spiked features on the vesicle surface (Figure S17). Energy dispersive spectroscopy (EDS) mapping and analysis were performed to confirm the elemental composition and spatial distribution of key components within P@Rg‐P_50_ (Figure 1C and Figure S18). The results successfully verified the co‐localization of expected elements from all constituent parts: the carbon, oxygen, nitrogen, phosphorus, and chlorine likely originate from PEAMP and R848, while the presence of sulfur and palladium could be ascribed to the methionine of gp100 peptides and the Chol‐Pd(II) complex of Chol‐PItEG_50_, respectively. The differential scanning calorimetry (DSC) thermograms and x‐Ray diffraction (XRD) diffractogram provided insight into the physical states and crystalline state of R848 or PEAMP, indicating the successful incorporation of R848 in PEAMP (Figure S19). The DLS analysis demonstrated that P@Rg‐P_50_ exhibit excellent stability for 7 days at 4°C with negligible changes in particle size and zeta potential (Figure 1D). However, upon exposure to esterase, the hydrodynamic diameters of P@Rg‐P_50_ undergo significant change owing to the esterase mediated hydrolysis on methacrylate of the hydrophobic shell of PEMAP, indicating the disaggregation of P@Rg‐P_50_ as observed in TEM images (Figure 1A and Figure S20). Drug release experiment further confirmed such results. As shown in Figure 1E, the drug release profile of R848 or gp100 peptides exhibited a sustained release pattern in the absence of esterase, with minimal drug release observed over 24 h. Conversely, in the presence of esterase, a rapid release of R848 or gp100 peptides was detected, confirming their ability to effectively release under the esterase‐rich environment of M2‐like TAMs [29]. Together, these results demonstrate that the obtained nanovesicles enable dual loading of gp100 and R848 with esterase‐responsive intracellular release, providing a basis to build a melanoma vaccine.

### 2.2 Mechanical Property of P‐P_m_ Nanovesicles

To investigate the impact of Chol‐PItEG_m_ decoration on vesicle rigidity, we prepared Chol‐PItEG_m_‐modified PEAMP nanovesicles without R848/gp100 loading (designated P‐P_m_) and characterized their mechanical properties using PeakForce atomic force microscopy in quantitative nanomechanical (QNM) mode. As shown in Figure 1F,G, the PEAMP and P‐P_m_ nanovesicles present as spherical entities with uniform size distribution. The atomic force microscopy (AFM) analysis reveals that the Young's modulus of PEAMP nanovesicles was 0.69 ± 0.05 GPa (Figure 1H), aligning with the reported nanovesicles mechanics [30]. Strikingly, Chol‐PItEG_m_ modification enhanced vesicle rigidity in a chain length‐dependent manner, with Young's modulus progressively increasing from 1.17 ± 0.15 GPa (P‐P_20_) to 1.97 ± 0.22 GPa (P‐P_30_) and 4.65 ± 0.17 GPa (P‐P_50_) (Figure 1H). The surface deformation results of P‐P_m_ are consistent with the mechanical properties observed in the Young's modulus analysis. The softer PEAMP exhibited a higher surface deformation of 8.14 ± 0.60 nm, whereas the stiffer P‐P_20_, P‐P_30_, and P‐P_50_ displayed lower surface deformations of 5.22 ± 0.33, 3.85 ± 0.97, and 1.44 ± 0.74 nm, respectively (Figure 1I). Such a phenomenon could be explained by the distinct contrast between the flexible mPEG_2000_ chains of PEAMP and the stereoregular helical structure of Chol‐PItEG_m_ on the surface of P‐P_m_. Specifically, PEAMP nanovesicles, which features flexible mPEG_2000_ chains in the outer layer, can readily adjust its spatial configuration when subjected to stress. Conversely, Chol‐PItEG_m_ on the surface of P‐P_m_ possess a stereoregular helical arrangement that imparts a higher resistance to conformational changes (Figure S21). Perspective from energy dissipation, when subjected to the applied force from the AFM tip, the softer PEAMP showed higher energy dissipation (16.66 ± 0.70 KeV) compared to the stiffer P‐P_20_, P‐P_30_, and P‐P_50_, which exhibited lower energy dissipation of 6.33 ± 0.25, 6.37 ± 0.32, and 1.55 ± 0.47 KeV, respectively (Figure 1J). This discrepancy arises because the softer PEAMP experience a prolonged contact time and a larger contact area under the applied force from the AFM tip, thus leading to increased energy dissipation. These results showed that the decoration of Chol‐PItEG_m_ chains significantly increased the rigidity of P‐P_m_, which was closely related to the chain length of Chol‐PItEG_m,_ demonstrating the successful fabrication of P‐P_m_ nanovesicles with tunable rigidity. Altogether, we confirm the successful preparation of rigidity‐tunable nanovesicles, which paves the way for downstream mechanistic and vaccination studies.

Further, given that the subsequent experiments were performed under physiological conditions, protein adsorption is an unavoidable event when nanovesicles encounter biological fluids. Accordingly, we examined whether serum exposure would alter the mechanical properties of the vesicles. As shown in Figure S22, incubation in 10% serum‐containing medium (SCM) did not cause any significant change in the size of the nanovesicles. Moreover, the rigidity‐related parameters, including modulus, deformation, and energy dissipation, remained comparable to those measured under serum‐free conditions. Collectively, these results suggest that possible adsorption of serum protein does not substantially compromise the mechanical integrity of the nanovesicles under the tested conditions.

### 2.3 Interactions Between P‐P_m_ and TAMs

Given macrophages are professional phagocytes, their initial physical engagement with particulate materials is a key trigger for downstream responses. We therefore quantified the interaction forces between P‐P_m_ nanovesicles and macrophages to determine whether vesicle stiffness dictates the strength of this initiating step. To eliminate the influence of biochemical signals from R848/gp100 peptides, a series of P‐P_m_ was examined. The cellular uptake assay indicates that the uptake of P‐P_50_ by RAW264.7 cells was 1.55‐fold, 1.29‐fold, and 1.07‐fold higher than that of softer PEAMP nanovesicles, P‐P_20_, and P‐P_30_, respectively, which is positively relevant to nanovesicles rigidity (Figure 2A and Figure S23). Confocal laser scanning microscopy (CLSM) images also supplied the coincident result that P‐P_50_ showed the highest fluorescence intensity within RAW264.7 cells compared to other groups at every time interval (Figure S24). We speculate that rigid nanoparticles (e.g., P‐P_50_, 4.65 ± 0.17 GPa) could maintain their shape well while soft nanoparticles (e.g., PEAMP with 0.69 ± 0.05 GPa) are more prone to undergo deformation, resulting in quite obvious difference in their phagocytosis [31, 32]. In other words, P‐P_m_ with higher rigidity form stronger interactions with macrophages, which is consistent with previous studies [33, 34]. To further identify the specific uptake pathways for P‐P_m_, pharmacological inhibitors were used to block distinct routes. As demonstrated in Figure 2B, the uptake mechanisms for P‐P_m_ appear to be the combination of clathrin‐mediated endocytosis and caveolae‐mediated endocytosis, as supported by the significant decrease in P‐P_m_ uptake by these two pathway inhibitors. This is reasonable since clathrin‐mediated endocytosis is the predominant pathway for internalizing a broad spectrum of molecules and transporters, whereas caveolae function as mechanosensors that respond to mechanical stress [35, 36, 37].

**FIGURE 2:**
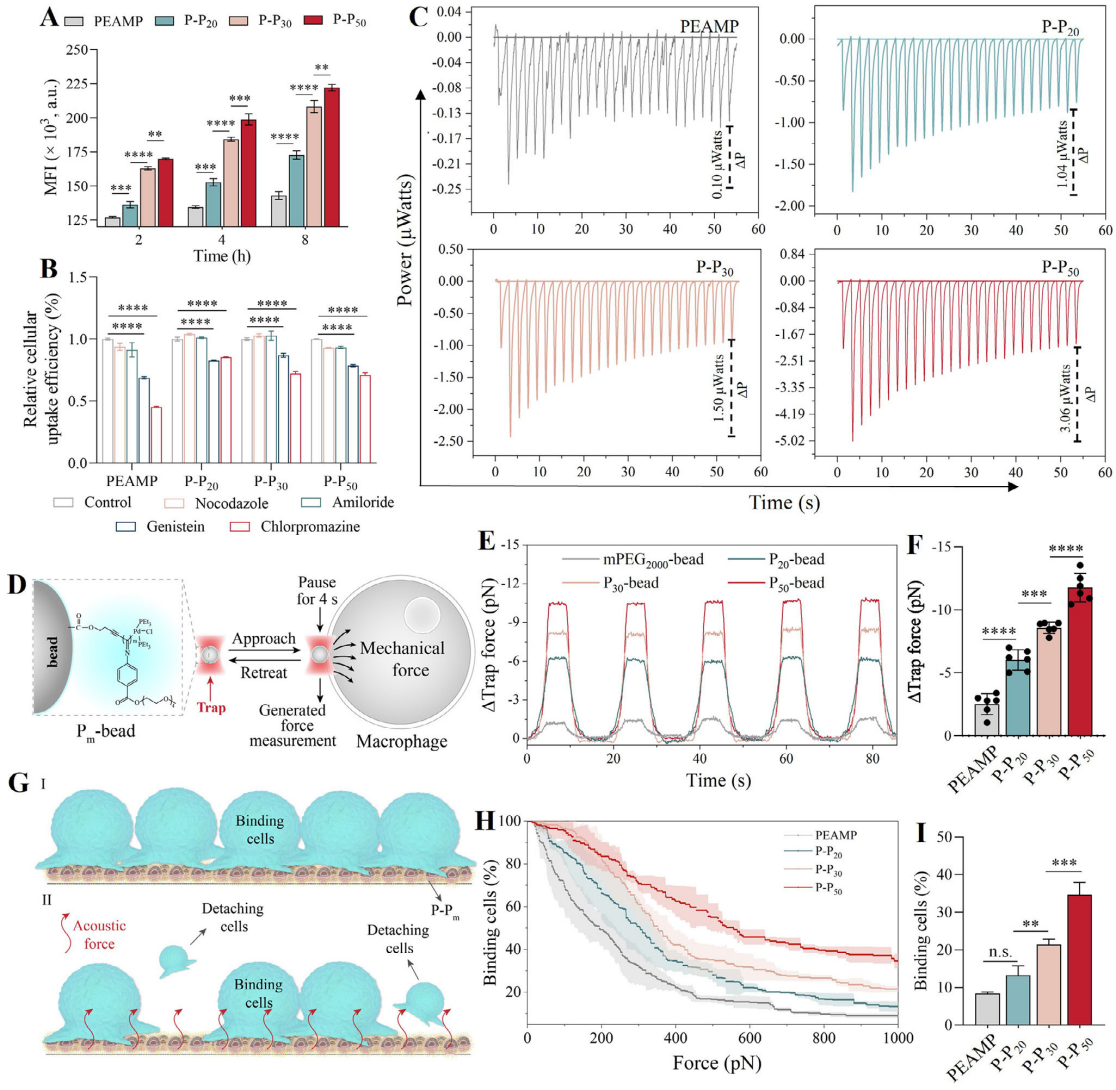
Interactions between macrophages and P‐P_m_. (A) Cellular uptake efficiency of PEAMP, P‐P_20_, P‐P_30_, and P‐P_50_ by RAW264.7 cells at 2, 4, and 8 h (*n* = 3). (B) Relative cellular uptake efficiency of P‐P_m_ by RAW264.7 cells pre‐treated with different endocytosis inhibitors for 2 h, data was normalized to a control that was not exposed to any inhibitors (chlorpromazine for clathrin‐mediated endocytosis inhibition:10 µg mL^−1^, genistein for caveolae‐mediated endocytosis inhibition: 100 µg mL^−1^, nocodazole for microtubule‐dependent endocytosis inhibition: 100 ng mL^−1^, and amiloride for micropinocytosis inhibition: 40 µg mL^−1^ (*n* = 3). (C) ITC curves showing the interactions between RAW264.7 cells and PEAMP, P‐P_20_, P‐P_30_, P‐P_50_. (D) Schematic depiction of the force measurement assay between P_m_‐beads and macrophage. The mPEG_2000_/HO‐PItEG_m_ decorated beads (denoted as mPEG_2000_‐beads/P_m_‐beads) were trapped and subjected to an approach‐pause‐retreat cycle for five‐time repeats. The forces generated during this motion were fully recorded, with baseline trap forces obtained by releasing bead and repeating the trajectory, subsequently subtracted for analysis (∆Trap force). Force is defined as positive when directed to the right. (E,F) Representative time trace of trap force and average trap force exerted by the P_m_‐beads on RAW264.7 cells during five measurement cycles (*n* = 6). (G) Schematic illustration of cell binding avidity assay measured by z‐Movi. Cells were interacted with P‐P_m_ pre‐coated on the chips for 2 min for subjected to increased acoustic force. (H,I) Percentage of binding cells with increasing amount of acoustic force (pN) and the percentage of binding cells at 1000 pN (*n* = 3). Data are presented as mean ± s.d. Statistical significance was determined by one‐way ANOVA with Tukey's multiple‐comparisons test. ^*^ *p* < 0.05, ^**^ *p* < 0.01, ^***^ *p* < 0.001, and ^****^ *p* < 0.0001; n.s., not significant.

Attachment of nanoparticles with cell surface occurs prior to cell uptake. Therefore, we next employed isothermal titration calorimetry (ITC) to investigate the molecular interactions between P‐P_m_ and RAW264.7 cells, offering deeper insights into their binding affinity and thermodynamic properties [38]. The binding affinity was evaluated by measuring the difference in power (∆P) between the maximum heat signal and the equilibrium state. It turned out that soft PEAMP nanovesicles did not induce significant thermal changes (∆p = 0.10 µWatts) (Figure 2C), suggesting a weak interaction with RAW264.7 cells. However, P‐P_50_ exerted the most significant heat generation during titration (∆p = 3.06 µWatts), which was 30.60‐fold, 2.94‐fold, and 2.04‐fold than that of PEAMP (∆p = 0.10 µWatts), P‐P_20_ (∆p = 1.04 µWatts), and P‐P_30_ (∆p = 1.50 µWatts), respectively. Collectively, these results indicate that P‐P_50_ exhibits the strongest binding affinity toward RAW264.7 cells among all tested formulations.

A single‐molecular optical tweezer fluorescence confocal system was further used for measuring the generated force of the bending cell membrane induced by P‐P_m_. Since single‐molecule optical tweezer systems operate at a microscale using intense laser beams to create optical traps for precise manipulation of individual molecules, the nanoscale P‐P_m_ cannot be directly utilized in this experiment. Herein, carboxylated microscale polystyrene beads (2 µm) modified with mPEG_2000_ or HO‐PItEG_m_ were prepared to mimic PEAMP or P‐P_m_ for the study. Briefly, HO‐PItEG_m_ were polymerized by using ItEG as a monomer and HO‐Pd(II) complex as an initiator instead of Chol‐Pd(II) complex for the polymerization of HO‐PItEG_m_ (Figures S25 and S26). Chemical structure and GPC analysis of HO‐PItEG_m_ were supplied in Figures S27 and S28 and Tables S4 and S5. FT‐IR spectra of P_m_‐beads indicate the successful modification of HO‐PItEG_m_ and mPEG_2000_ (Figures S29 and S30). Figure 2D provides an overview of the experimental procedure. Typically, mPEG_2000_‐beads or P_m_‐beads were trapped and subjected to an approach‐pause‐retreat cycle for five‐time repeats. The forces generated during this motion were fully recorded and subsequently subtract the baseline trap forces for analysis (∆Trap force). As shown in Figure 2E and Movie S1, as the trapped bead approach the target cell, the generated forces gradually increased until they contacted. At this state, the generated force represents the interaction between the P_m_‐bead and the cell. The P_50_‐beads were observed to exert the highest force of 11.77 ± 1.04 pN toward macrophages, significantly surpassing the forces exerted by softer mPEG_2000_‐beads, P_20_‐beads, and P_30_‐beads of 2.52 ± 0.77 pN, 6.02 ± 0.74 pN, and 8.58 ± 0.41 pN, respectively (Figure 2E,F). These results provide compelling evidence that P_50_‐beads with stronger mechanical strength elicited more substantial forces with macrophage.

Furthermore, the cell binding assay also confirmed the same conclusion. As shown in Figure 2G, RAW264.7 cells were co‐incubated with P‐P_m_ and gradually detached under the applied acoustic force. It was observed that when the acoustic force reached 1000 pN, 8.41% ± 0.32% of the cells bound to PEAMP remained attached, whereas for P‐P_20_, P‐P_30_, and P‐P_50_, the retention percentages were 13.28% ± 2.03%, 21.47% ± 1.16%, and 34.59% ± 2.75%, respectively (Figure 2H,I). These results emphasize the strong interaction between RAW264.7 cells and P‐P_50_, highlighting the potential role of nanoparticle rigidity in influencing cellular interactions and subsequent cellular uptake.

### 2.4 M2‐Like Macrophage Repolarization

To assess whether mechanical stimulation confers adjuvanticity, we next quantified macrophage repolarization and antigen presentation effects by P@Rg‐P_m_. The potency of P@Rg‐P_m_ with tunable rigidity in M2‐like macrophage repolarization was emphatically investigated (Figure 3A). M2‐like RAW264.7 cells were obtained by treating M0‐like RAW264.7 cells with IL‐4 for 24 h, as confirmed by increased CD206 expression and IL‐10 secretion (Figure S31). The negligible cytotoxicity of Chol‐PItEG_m_ was confirmed in multiple cell lines (including RAW264.7, HUVEC, L929, and C166 cells) (Figure S32). The M2‐like cells were then exposed to various nano‐agents for an additional 24 h (Figure 3O). Distinct morphological differences were observed between macrophage phenotypes: M1‐like macrophages (induced by LPS + IFN‐γ) exhibited characteristic pseudopodia formation and increased cell area, while M2‐like macrophages (induced by IL‐4) displayed a rounded morphology with reduced cell area. Comparative analysis revealed that the highest‐rigidity P@Rg‐P_50_ nanovesicles treatment most effectively recapitulated the M1‐like morphological shift (Figure 3B,C). Flow cytometry analysis corroborated these morphological observations by revealing that P@Rg‐P_50_ nanovesicles significantly upregulated M1 markers (CD80/CD86/MHC II) while downregulating M2 marker CD206 in M2‐like RAW264.7 cells (Figure 3D–G and Figure S33). Similar patterns were observed in the secretion of proinflammatory cytokines of TNF‐α, IL‐1β, and IL‐12 (Figure 3H–J). Correspondingly, the expression of key mRNAs, including TNF‐α, IL‐1β, IL‐12, and iNOS, further substantiated the potency of P@Rg‐P_m_ nanovesicles in modulating macrophage phenotypes (Figure 3K–N). Furthermore, bone marrow‐derived macrophages (BMDMs) were harvested (Figure S34) and employed as a cellular model for a parallel investigation. Excitingly, P@Rg‐P_50_ exhibited remarkable reprogramming capacity in BMDMs, supported by significant alterations in surface marker expression of CD80/CD86/MHC II/CD206 (Figure S35), along with a marked increase secretion of TNF‐α, IL‐1β, and IL‐12 (Figure S36). Collectively, our data show that the highest‐rigidity P@Rg‐P_50_ nanovesicles induce macrophage repolarization through mechanical stimulation, thereby exhibiting pronounced adjuvanticity.

**FIGURE 3:**
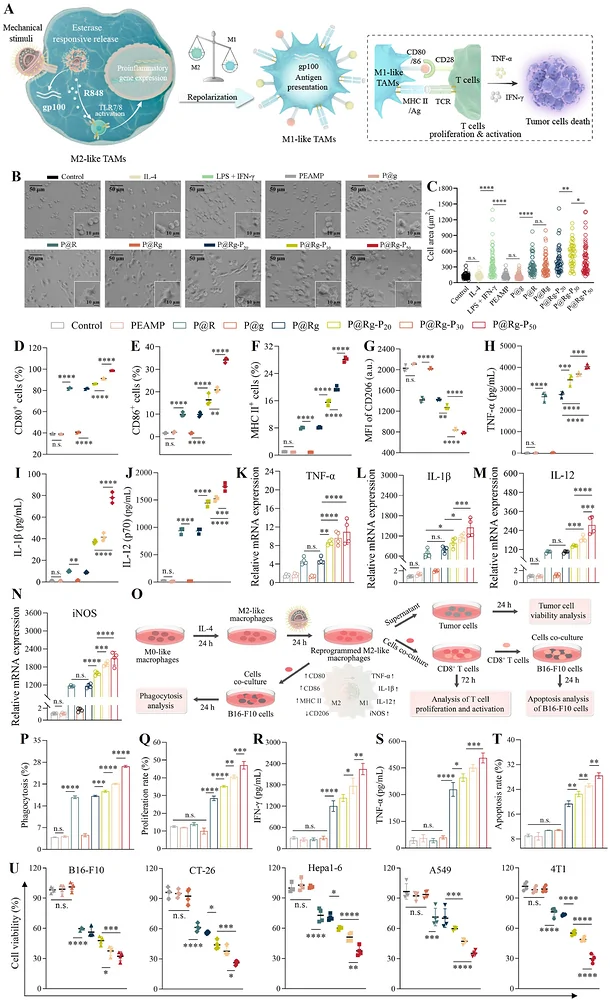
M2‐like macrophage repolarization by rigid P@Rg‐P_m_ and the resulting CD8^+^ T cells activation potential. (A) Scheme illustration of reprogranmed‐M2‐like TAMs by P@Rg‐P_m_ and their CD8^+^ T cells activation potential. (B,C) Optical morphology images and cell areas (determined by Image J software) of RAW264.7 cell after indicated treatment for 18 h (Control, *n* = 88. IL‐4, *n* = 69. LPS + IFN‐γ, *n* = 73. PEAMP, *n* = 95. P@R, *n* = 57. P@g, *n* = 129. P@Rg, *n* = 53. P@Rg‐P_20_, *n* = 47. P@Rg‐P_30_, *n* = 43. P@Rg‐P_50_, *n* = 60), scale bar: 10 µm. (D–G) The surface markers expression analysis of CD80, CD86, MHC II, and CD206 in M2‐like RAW264.7 cells after the indicated treatment for 24 h (*n* = 3). (H–J) Cytokines detection of TNF‐α, IL‐1β, and IL‐12p70 in the culture supernatant of M2‐like RAW264.7 cells after indicated treatment for 24 h (*n* = 3). (K–N) Relative mRNA expression of TNF‐α, IL‐1β, IL‐12, and iNOS in M2‐like RAW264.7 cells after indicated treatment for 24 h (*n* = 4). (O) Flowchart of the assay. (P) Phagocytosis rate of B16‐F10 cells by various nano‐agents reprogrammed M2‐like RAW264.7 cells for 24 h (*n* = 3). (Q) Proliferation rate of CD8^+^ T cell after co‐culture with various nano‐agents reprogrammed‐M2‐like BMDMs for 72 h (*n* = 3). (R,S) Cytokine secretion of IFN‐γ and TNF‐α of CD8^+^ T cells after co‐culture with various nano‐agents reprogrammed M2‐like BMDMs for 72 h (*n* = 3). (T) Apoptosis rate of B16‐F10 cells after co‐culture with CD8^+^ T cells activated by various nano‐agents reprogrammed M2‐like BMDMs for 24 h (*n* = 3). (U) Cell viability analysis of B16‐F10, CT‐26, Hepa1‐6, A549, and 4T1 cells treated with the supernatant of various nano‐agents reprogrammed M2‐like RAW264.7 cells for 24 h (*n* = 4). Data are presented as mean ± s.d. Statistical significance was determined by one‐way ANOVA with Tukey's multiple‐comparisons test. ^*^ *p* < 0.05, ^**^ *p* < 0.01, ^***^ *p* < 0.001, and ^****^ *p* < 0.0001; n.s., not significant.

### 2.5 Enhanced Tumor Cell Inhibition

As phagocytosis is a key effector function of macrophages that underlies their roles in tumor cell clearance, we further evaluated their phagocytic capacity. As shown in Figure 3P and Figure S37A, co‐culturing re‐polarized macrophages with B16‐F10 cells demonstrated that macrophages treated with P@Rg‐P_50_ exhibited the most pronounced phagocytic activity, with a phagocytosis rate of 26.69% against B16‐F10 cells. Next, the supernatant of P@Rg‐P_m_‐treated M2‐like macrophages was collected and cultured with B16‐F10, CT‐26, Hepa1‐6, A549, and 4T1 cells for 24 h. Cell counting kit‐8 (CCK‐8) assay revealed that the supernatant from P@Rg‐P_50_‐treated M2‐like macrophages exhibit the most prominent suppressive effect on these cells (Figure 3U). Importantly, it was found that P@Rg‐P_m_ did not directly inhibit tumor cell growth (Figure S38), indicating the cytotoxicity was attributed to the cytokines secreted by the P@Rg‐P_m_‐treated M2‐like macrophages. Furthermore, the transwell co‐culture system was used to reconfirm this inhibitory effect of P@Rg‐P_m_‐treated M2‐like macrophages on B16‐F10 cells. The least carboxyfluorescein succinimidyl ester (CFSE) fluorescence decreases suggest the most potent inhibition effect of P@Rg‐P_50_ on B16‐F10 cell proliferation (Figure S39).

As professional antigen‐presenting cells, the antigen cross‐presentation capacity of TAMs plays a crucial role in activating CD8^+^ T cells and orchestrating immune responses [39, 40]. Therefore, we also investigated the gp100 peptide processing and presentation capabilities of M2‐like macrophages reprogrammed with various nano‐agents (Figure 3O). Figure 3F shows the increased MHC II expression in RAW264.7 cells by P@R compared to PEAMP and P@g due to R848 bioactivity, the further MHC II enhancement was observed as the rigidity of P@Rg‐P_m_ increased. A similar phenomenon occurred in BMDMs with the strongest MHC II expression in P@Rg‐P_50_ group (Figure S35C). These results confirm that the rigid P@Rg‐P_50_ nanovesicles to improve the antigen presenting function of TAMs. As depicted in Figure 3Q and Figure S37B, PEMAP, P@R, or P@g‐treated M2‐like macrophages exerted minimal effects on CD8^+^ T cell proliferation (11.97%, 13.85%, and 9.95%, respectively). In contrast, P@Rg‐treated M2‐like macrophages markedly promoted CD8^+^ Tcell proliferation (28.41%), owing to their acquisition of M1‐like phenotypic functions after R848‐induced repolarization, which endowed them with enhanced co‐stimulatory signaling and more efficient antigen presentation. Remarkably, P@Rg‐P_50_‐treated M2‐like macrophages significantly enhanced CD8^+^ T cell proliferation by 46.84% and robustly promoted IFN‐γ and TNF‐α secretion, highlighting their superior capacity to activate CD8^+^ T cell responses and drive adaptive immunity (Figure 3R,S). The activated CD8^+^ T cells were then co‐cultured with B16‐F10 cells to evaluate their apoptosis activity (Figure 3T and Figure S37C). Due to the suboptimal activation of CD8^+^ T cells by PEMAP, P@R, or P@g‐treated M2‐like macrophages, CD8^+^ T cells in these groups did not exhibit significant cytotoxicity to B16‐F10 cells (8.90%, 10.79%, and 10.88%, respectively). However, owing to the pronounced activation of P@Rg‐P_50_‐treated M2‐like macrophages on CD8^+^ T cells, these CD8^+^ T cells demonstrated the most potent induction of apoptosis in B16‐F10 cells (28.45%). Parallel experiments conducted with 4T1 cells as alternative targets revealed that the apoptosis of P@Rg‐P_m_‐stimulated CD8^+^ T cells was comparatively modest against 4T1 cells compared to their effect on B16‐F10 cells (Figure S40). Collectively, these findings establish that P@Rg‐P_50_ confers mechanically induced adjuvanticity in TAMs, as evidenced by enhanced antigen‐presenting capacity and consequent anti‐tumor immunity.

### 2.6 P‐P_m_ Promotes the Activation of YAP/TAZ and Piezo1

We next sought to elucidate the signaling pathways underlying the adjuvant properties of rigid P‐P_m_. Yes‐associated protein (YAP) and transcriptional coactivator with PDZ‐binding motif (TAZ) are known as key players in cellular mechanotransduction [41]. Nuclear translocation of YAP/TAZ signifies a crucial cellular response to mechanical cues, indicating that the cell is actively interpreting and responding to mechanical stimuli from its environment. Immunofluorescence staining of RAW264.7 cells treated with P‐P_m_ revealed that YAP/TAZ primarily localized in the cytoplasm of macrophages with a nuclear localization rate of 27.18% ± 4.20% under basal conditions, and the addition of PEAMP did not significantly alter this distribution (30.59% ± 1.92%) (Figure 4A–C). In marked contrast, the introduction of P‐P_m_ induced significant alterations in YAP/TAZ nuclear translocation, with the most pronounced effects observed for the P‐P_50_ (80.55% ± 3.13%). This difference is likely due to the substantial mechanical stimuli exerted by P‐P_50_, which triggers a more pronounced mechanotransductive response of the YAP/TAZ pathway in macrophages. The pronounced overlap in gray values between the nucleus and YAP/TAZ (Figure 4A) also corroborated by the protein and mRNA expression of YAP1 (Figure 4D and Figure S41A). Additionally, verteporfin, a pharmacological inhibitor of YAP1, was used to block the mechanotransduction pathway mediated by YAP1. The results showed that verteporfin treatment significantly suppressed the expression of M1‐like markers induced by P‐P_m_ in M2‐like macrophages, including CD80, CD86, and MHC II (Figure S41B–D). Hence, YAP/TAZ may serve as a pivotal mediator through which P‐P_m_ mechanically instigates the repolarization and improves antigen presenting capability of M2‐like macrophage phenotypes (Figure S41E).

**FIGURE 4:**
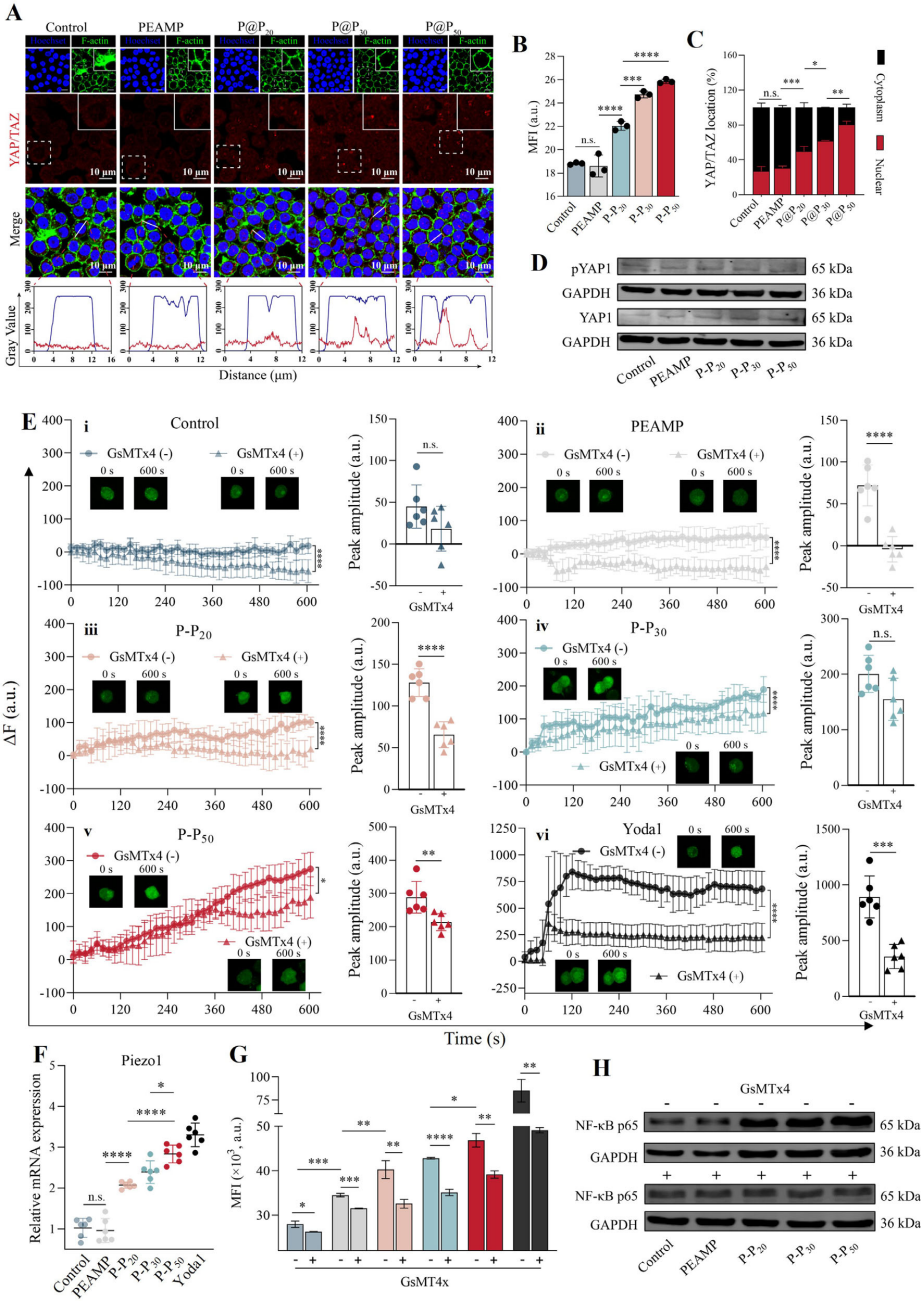
Activation of YAP/TAZ and Piezo1 by P‐P_m_. (A) Immunofluorescence confocal images of YAP/TAZ in RAW264.7 cells exposed to PEAMP, P‐P_20_, P‐P_30_, and P‐P_50_ for 4 h, the intensity of gray values along the white line from left to right in the corresponding image was delineated, blue for the nucleus and red for YAP/TAZ, scale bar: 10 µm. (B) Mean fluorescence intensity of YAP/TAZ in RAW264.7 cells exposed to PEAMP, P‐P_20_, P‐P_30_, and P‐P_50_ for 4 h (*n* = 3). (C) Quantification of nuclear to cytoplasmic ratios of YAP/TAZ in RAW264.7 cells exposed to PEAMP, P‐P_20_, P‐P_30_, and P‐P_50_ for 4 h (*n* = 3). (D) WB analysis of YAP and pYAP, GAPDH proteins expression in RAW264.7 cells treated with PEAMP, P‐P_20_, P‐P_30_, and P‐P_50_ for 4 h. (E) Time trace of Flou‐4 fluorescence intensity and relative peak amplitude detected by CLSM in RAW264.7 cells treated with PBS (as control), PEAMP, P‐P_20_, P‐P_30_, P‐P_50_, and Yoda1 (5 µM) with or without GsMTx4 (3 µm, 0.5 h) pre‐treatment (*n* = 6). Real‐time imaging was conducted for capturing images every 15 s for a 10 min period. ΔF representing the change of Flou‐4 fluorescence intensity. The inserted photos represent the fluorescence intensity of macrophages in 0 or 600 s. (F) Relative mRNA expression of Piezo1 in RAW264.7 cells exposed to PEAMP, P‐P_20_, P‐P_30_, and P‐P_50_ for 24 h (*n* = 6). (G) Flou‐4 fluorescence intensity of RAW264.7 cells treated with PBS (as control), PEAMP, P‐P_20_, P‐P_30_, P‐P_50_, and Yoda 1 (5 µm) for 2 h with or without GsMTx4 (3 µm, 0.5 h) pre‐treatment (*n* = 3). (H) WB analysis of NF‐κB p65 and GAPDH proteins expression in RAW264.7 cells treated with PEAMP, P‐P_20_, P‐P_30_, and P‐P_50_ for 24 h with or without GsMTx4 (3 µm) pre‐treatment. Data are presented as mean ± s.d. Statistical significance was determined by one‐way ANOVA with Tukey's multiple‐comparisons test. ^*^ *p* < 0.05, ^**^ *p* < 0.01, ^***^ *p* < 0.001, and ^****^ *p* < 0.0001; n.s., not significant.

Mechanically activated ion channel Piezo1 has earned most of its fame for its remarkable contributions to mechanotransduction in various physiological and pathological processes [42, 43, 44]. The correlation between the stiffness sensitivity of macrophage and Piezo1 has also been recognized [45]. Accordingly, we are intrigued to explore whether the mechanical stimulation of macrophages by rigid P‐P_m_ also triggers the activation of the mechanosensitive ion channel Piezo1. It was known that Piezo1 ion channel exhibits a high affinity for calcium ions (Ca^2+^), allowing for a significant influx of Ca^2+^ upon activation to regulate various physiological activities within the cell [46, 47]. Therefore, Fluo‐4 acetoxymethyl ester (Fluo‐4, AM) probe was used to visualize Ca^2+^ influx in macrophages stimulated by P‐P_m_. Quantifying the changes in Fluo‐4 fluorescence signal (∆F) allows for the determination of intracellular Ca^2+^ influx levels. As depicted in Figure 4E(vi) and Movie S7, macrophages treated with Yoda1 (specific Piezo1 agonist) exhibited a sharp increase in Flou‐4 fluorescence intensity due to rapid intracellular Ca^2+^ influx, suggesting the presence of functional Piezo1 channels on the macrophages. Notably, macrophages treated with P‐P_50_ demonstrated the most robust Ca^2+^ influx signal compared to the groups treated with PBS, PEAMP, P‐P_20_, or P‐P_30_, as evidenced by the highest peak amplitude of 288.7 ± 43.4 (Figure 4E(v) and Movie S6). In contrast, the peak amplitudes of Ca^2+^ signals induced by PEAMP, P‐P_20_, and P‐P_30_ were significantly lower, recorded at 72.1 ± 22.3, 128.1 ± 15.2, and 200.7 ± 30.5, respectively (Figure 4E(i–iv) and Movies S2–S5). This observation underscores that P‐P_50_, with the highest mechanical stimulation, is the most effective at activating Piezo1 channel opening. The mRNA expression analysis of Piezo1 corroborated this observation (Figure 4F). Moreover, we unexpectedly obtained compelling visual evidence showing that macrophages exhibit a stronger interaction with P‐P_m_ particles of higher mechanical rigidity. Videos show that macrophages in the P‐P_50_‐treated group demonstrated continuous pseudopodia extension and vigorous internalization activity, suggesting a significantly more pronounced uptake of P‐P_50_ particles (Movie S6), whereas the cellular response to the softer PEAMP particles remained markedly subdued (Movie S3).

To further confirm the specificity of rigid P‐P_m_ for the Piezo1 ion channel, GsMTx4, a reversible inhibitor known to target the Piezo1 channel, was used to block Piezo1 activity on macrophages. As shown in Figure 4E(i–vi) and Movies S2–S7, pre‐treatment with GsMTx4 markedly attenuated the Ca^2+^ influx elicited by various nano‐agents and Yoda1. Flow cytometry results also confirmed that the enhanced Ca^2+^ signaling triggered by P‐P_m_ was difficult to sustain in the presence of GsMTx4 (Figure 4G and Figure S42). These results implied that the activation of mechanosensitive pathways by rigid P‐P_m_ in macrophage is highly associate with Piezo1 channels. Moreover, the NF‐κB signaling pathway is known to essential in modulating macrophage inflammatory responses. The activation of NF‐κB signaling pathway is regarded closely correlated with intracellular Ca^2+^ concentrations [48]. Thus, we hypothesize that the calcium influx induced by Piezo1 stimulation via P‐P_m_ may further trigger the activation of the NF‐κB signaling pathway. As illustrated in Figure 4H, the western blot analysis reveals that P‐P_m_ significantly upregulates the protein expression of NF‐κB p65. However, this upregulation is markedly attenuated by GsMTx4 treatment, likely due to the inhibition of Ca^2+^ influx mediated by GsMTx4 [45, 49, 50]. Taken together, these findings demonstrate that rigid P‐P_m_ nanovesicles activate mechanotransduction signaling pathways, providing insight into the mechanically induced adjuvanticity of P‐P_m_ in TAMs.

### 2.7 Therapeutic Benefit for B16‐F10 Murine Melanoma

We next assessed whether P@Rg‐P_50_ realizes mechanically induced adjuvanticity in vivo by reversing TAMs phenotypes and reshaping the tumor immune microenvironment. B16‐F10 tumor‐bearing C57BL/6 mice were randomized into six groups and treated with Saline, PEAMP, P‐P_50_, free R848 + gp100 peptides (termed as Free Rg), P@Rg, or P@Rg‐P_50_ every three days for five times (Figure 5A). As shown in Figure 5B–E and Figure S43A, it is evident that B16‐F10 cells grew aggressively in saline and PEAMP treated group mice. P‐P_50_ demonstrated a modest inhibition rate of 11.20%, indicating its potential for immune modulation. In addition, Free Rg and P@Rg‐treated group mice showed stronger tumor regression with tumor inhibition rate of 64.78% and 65.72%, respectively, partly due to the biochemical immune activation induced by R848. Remarkably, the most substantial action was observed in P@Rg‐P_50_‐treat group with 87.85% tumor inhibition rate, which is signally higher than that of P@Rg, indicating the positive attribution of mechanically induced adjuvanticity. Similarly, the hematoxylin and eosin (H&E) staining results reveal that tumor cells in the P@Rg‐P_50_ treatment group exhibit a more dispersed arrangement, with condensed nuclei and abundant vacuolar structures, suggesting more necrotic cells (Figure S44). Meanwhile, the increased terminal deoxynucleotidyl transferase dUTP nick end labeling (TUNEL)‐positive and decreased Ki67‐positive staining demonstrate the remarkable anti‐tumor effect of P@Rg‐P_50_ (Figure S44). Notably, the treatment almost did not induce any severe systemic adverse effects throughout the study, as evidenced by the stable body weight, unaltered histopathology analysis of major organs and normal levels of alanine aminotransferase (ALT), aspartate aminotransferase (AST), blood urea nitrogen (BUN), and creatinine (CREA) (Figures S43B, S45 and S46).

**FIGURE 5:**
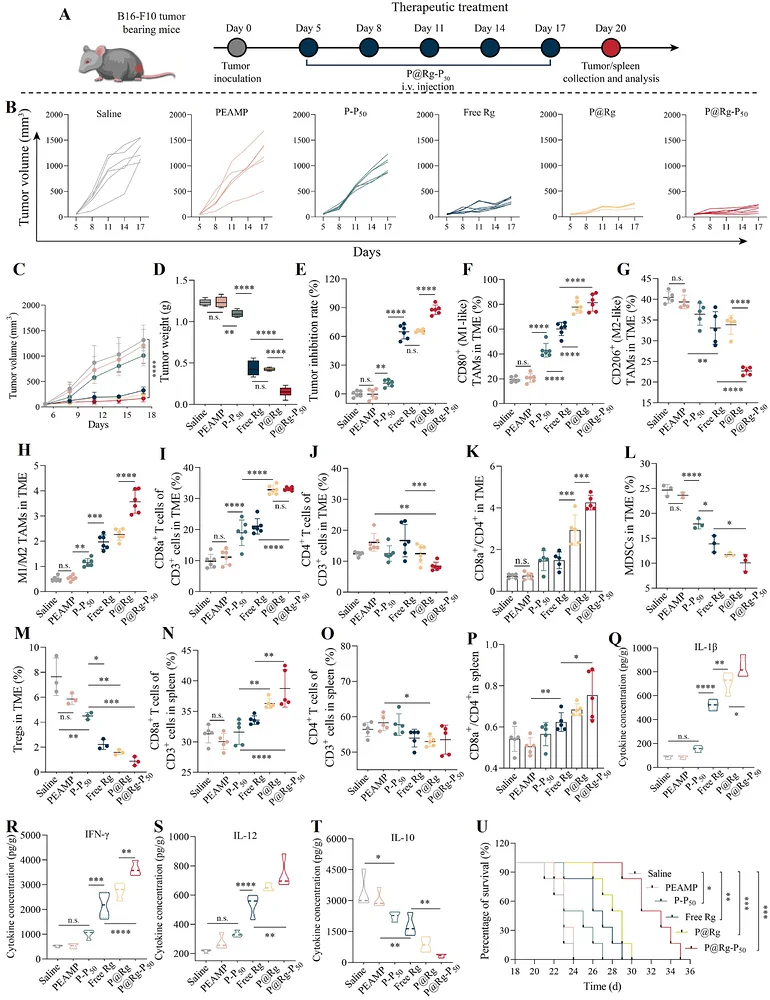
TAMs reprograming and immunosuppressive TME alleviation potential of P@Rg‐P_50_ in B16‐F10 murine melanoma model. (A) Scheme of B16‐F10 tumor mouse model for examining the antitumor immune response of Saline, PEAMP, P‐P_50_, Free Rg, P@Rg, or P@Rg‐P_50_. (B,C) Individual tumor growth curves and average tumor growth curves of B16‐F10 tumor‐bearing mice treated with indicated nano‐agents (*n* = 6). (D,E) Tumor weight and tumor inhibition rates of B16‐F10 tumor‐bearing mice after indicated treatment (*n* = 6). (F,G) Percentage of CD80^+^ (M1‐like) TAMs (*n* = 6) and CD206^+^ (M2‐like) TAMs in TME (*n* = 5). (H) Ratios of M1/M2 like TAMs (*n* = 6). (I) Percentage of CD8a^+^ T cells in TME (*n* = 6). (J) Percentage of CD4^+^ T cells in TME (*n* = 6). (K) Ratios of CD8^+^ T cells and CD4^+^ T cells in TME of B16‐F10 tumor‐bearing mice (*n* = 5). (L) Percentage of MDSCs in TME (*n* = 3). (M) Percentage of Tregs in TME (*n* = 3). (N) Percentage of CD8^+^ T cells in spleen (*n* = 5). (O) Percentage of CD4^+^ T cells in spleen (*n* = 5). (P) Ratios of CD8^+^ T cells and CD4^+^ T cells in spleen (*n* = 5). (Q–T) Cytokine concentration of IL‐1β, IFN‐γ, IL‐12, and IL‐10 in TME (*n* = 4), the width represents kernel density estimation, the center line marks the median. (U) Survival curves of B16‐F10 tumor‐bearing mice after indicated treatment (*n* = 6). Data are presented as mean ± s.d. Statistical significance was determined by one‐way ANOVA with Tukey's multiple‐comparisons test. ^*^ *p* < 0.05, ^**^ *p* < 0.01, ^***^ *p* < 0.001, and ^****^ *p* < 0.0001; n.s., not significant.

We next evaluated the impact of P@Rg‐P_50_ on immune cell populations within the TME using flow cytometry. We first investigated whether the nanoparticles could be internalized by TAMs in vivo. Flow cytometry analysis of tumor single‐cell suspensions revealed that both PEAMP@Ce6 and P‐P_50_@Ce6 exhibited higher intracellular fluorescence signals in TAMs than free Ce6 (Figure S47). Notably, P‐P_50_@Ce6 showed the highest MFI, indicating superior uptake of the rigid nanoparticles by TAMs. These results confirmed the successful accumulation of P‐P_50_ within tumors and its direct internalization by TAMs, providing a cellular basis for subsequent macrophage reprogramming in TME. The impact of P@Rg‐P_50_ on immune cell populations within the TME were illustrated in Figure 5F–P. Compared to the P@Rg‐treated group, P@Rg‐P_50_ treatment dramatically reshaped the TME by causing a modest increase in the proportion of M1‐like TAMs (F4/80^+^CD11b^+^CD80^+^) (77.9% vs. 81.4%) and profoundly suppressing M2‐like TAMs (F4/80^+^CD11b^+^CD206^+^) (33.9% vs. 22.7%) (Figure 5F,G). This striking shift resulted in a sharp increase in the M1/M2 ratio (2.3% vs. 3.6%) (Figure 5H). Moreover, the reversal of the M2‐like TAMs induced by P@Rg‐P_50_ yielded broader implications for the immune landscape within the TME, facilitating infiltration and activation of various immune cell populations. As illustrated in Figure 5I–K, compared to P@Rg‐treated group, P@Rg‐P_50_ treatment effectively increased the CD8^+^/CD4^+^ T cell ratio (CD45^+^ CD3^+^ CD8^+^/CD45^+^ CD3^+^ CD4^+^) (2.9 vs. 4.3). This elevated ratio, which serves as a crucial prognostic indicator for immunotherapy efficacy, reflects a robust enhancement of CD8^+^ T cell‐mediated anti‐tumor immunity [51]. Additionally, the levels of immunosuppressive cells within the TME were investigated, specifically focusing on myeloid derived suppressor cells (MDSCs) (CD45^+^ Gr‐1^+^ CD11b^+^) and regulatory T cells (Tregs) (CD4^+^ CD25^+^ Foxp3^+^), as they play a pivotal role in tumor immune evasion and resistance to immunotherapy. As expected, following the P@Rg‐P_50_‐treatment, a notable reduction in the subsets of MDSCs and Tregs was observed, suggesting an alleviation in immune system suppression (Figure 5L,M). Complementary findings from immune cell analysis within the spleen further support these observations. As depicted in Figure 5N–P, P@Rg‐P_50_ treatment led to significant enhancements in both the proportion of CD8^+^ T cells and the CD8^+^/CD4^+^ T cell ratio in spleen. The enrichment of CD8^+^ T cells in the spleen further confirms the activation of the overall immune response. Cytokine levels in the TME were assessed. P@Rg‐P_50_ evoked the highest level of proinflammatory factors generation including IL‐1β, IFN‐γ, and IL‐12, which collectively contribute to the enhancement of anti‐tumor immunity by promoting inflammation and activating immune cells (Figure 5Q–S). Notably, a decrease in IL‐10 was also observed (Figure 5T), which is particularly critical given that M2‐like TAMs are the primary producers of this immunosuppressive cytokine within TME [52]. Survival analysis revealed that administration of P@Rg‐P_50_ significantly extended the survival period of B16‐F10 tumor‐bearing mice (Figure 5U). Taken together, these results demonstrated the potential of P@Rg‐P_50_ with adjuvanticity of P‐P_50_ in reshaping the immune landscape within the TME through the reprogramming of M2‐like TAMs and the activation of anti‐tumor immunology via gp100 antigen.

### 2.8 Long‐Term Immune Protection for B16‐F10 Murine Melanoma

Encouraged by the beneficial effects of P@Rg‐P_50_ in suppressing tumor growth, the tumor prevention potential of P@Rg‐P_50_ was investigated. Naïve C57BL/6 mice were randomized into six groups and immunized with Saline, PEAMP, P‐P_50_, Free Rg, P@Rg, or P@Rg‐P_50_ every three days for five times (Figure 6A). The B16‐F10 cells then inoculated in the right hind legs of C57BL/6 mice, and the tumor growth were monitored in the following two weeks. As shown in Figure 6B–F, mice immunized with PEAMP or P‐P_50_ exhibited rapid tumor growth. Free Rg and P@Rg displayed slower tumor growth rate with final tumor inhibition rate of 67.11% and 75.33%, respectively. Notably, P@Rg‐P_50_ vaccination demonstrated pronounced preventive efficacy, significantly reducing B16‐F10 tumor weight by 89.11%. This remarkable outcome is likely attributable to the immune activation induced by the TLR7/8 agonist R848 and P‐P_50_, coupled with the antigen‐specific immune response triggered by the gp100 peptides. Moreover, the formation of immunological memory is a critical factor contributing to the prevention efficacy of P@Rg‐P_50_. The serum antibody analysis corroborates this notion as depicted in Figure 6G and Figure S48, where P@Rg‐P_50_ significantly boosted antibody production of both IgG1 and IgG2a. This robust and antigen‐specific immune response induced by the P@Rg‐P_50_ vaccine enhances the immune system's capacity to swiftly recognize and destroy tumor cells upon subsequent exposures. Significantly, the more substantial increase in IgG2a levels indicates that P@Rg‐P_50_ predominantly drove a Th1‐skewed immune response. This type of response is essential to establish enduring immunological memory, which is vital for long‐term protection against tumor re‐emergence [53].

**FIGURE 6:**
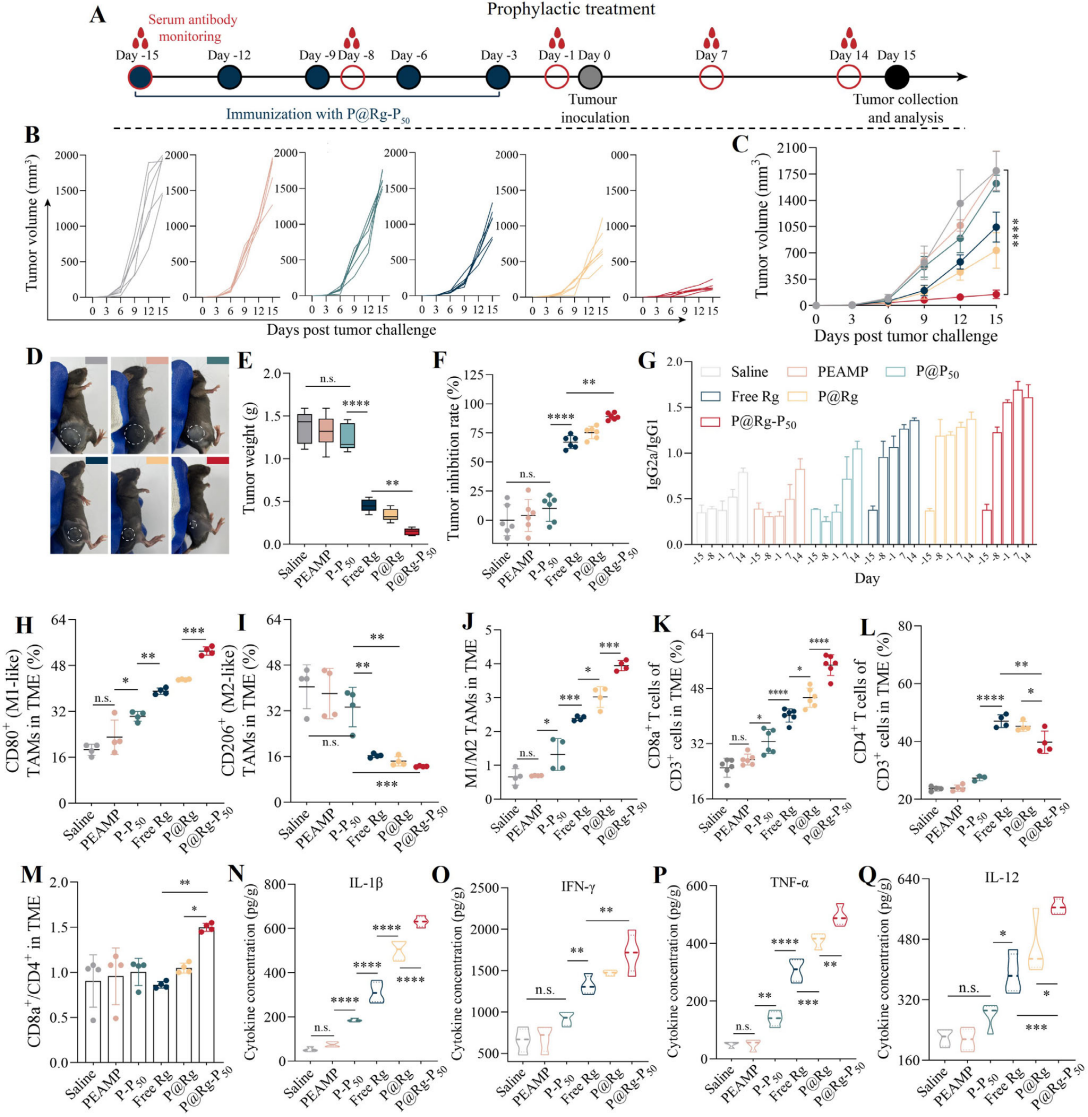
Prolonged immunological defense by P@Rg‐P_50_ in B16‐F10 murine melanoma model. (A) Scheme of B16‐F10 mice tumor model for examining prolonged immunological defense of Saline, PEAMP, P‐P_50_, Free Rg, P@Rg, or P@Rg‐P_50_. (B,C) Individual tumor growth curves and average tumor growth curves of B16‐F10 tumor‐bearing mice treated with indicated nano‐agents (*n* = 6). (D) Representative tumors of mice at day 10. (E,F) Tumor weight and tumor inhibition rate of B16‐F10 tumor‐bearing mice after indicated treatment (*n* = 6). (G) Serum IgG2a/IgG1 antibody level of mice treated with indicated nano‐agents (*n* = 3). (H,I) Percentage of M1‐like (CD80^+^) TAMs (*n* = 4) and M2‐like (CD206^+^) TAMs (*n* = 4) in TME. (J) Ratios of M1/M2 like TAMs (*n* = 4). (K) Percentage of CD8^+^ T cells in TME (*n* = 6). (L) Percentage of CD4^+^ T cells in TME (*n* = 4). (M) Ratios of CD8^+^ T cells and CD4^+^ T cells in TME of B16‐F10 tumor‐bearing mice treated with Saline, PEAMP, P‐P_50_, Free Rg, P@Rg, or P@Rg‐P_50_ in prophylactic treatment (*n* = 4). (N–Q) Cytokine concentration of IL‐1β, IFN‐γ, TNF‐α, and IL‐12 (*n* = 4), the width represents kernel density estimation, the center line marks the median. Data are presented as mean ± s.d. Statistical significance was determined by one‐way ANOVA with Tukey's multiple‐comparisons test. ^*^ *p* < 0.05, ^**^ *p* < 0.01, ^***^ *p* < 0.001, and ^****^ *p* < 0.0001; n.s., not significant.

We further assessed the composition of various immune cells in the TME of B16‐F10 tumors. Interestingly, although P‐P_50_ exhibited limited prevention effect on B16‐F10 tumor progression, flow cytometry analysis indicated its potential to induce immune activation. As shown in Figure 6H, P‐P_50_ immunization resulted in an increase in M1‐like TAMs within the B16‐F10 TME compared with the saline group (30.34% vs. 18.68%). Both the Free Rg and P@Rg immunization groups clearly exhibited a more favorable immune microenvironment, characterized a decrease in M2‐like TAMs (16.35% and 14.40%, respectively) (Figure 6I) and an increase in the M1/M2 TAMs ratio (2.4 and 3.0, respectively) (Figure 6J). Notably, compared with P@Rg, the P@Rg‐P_50_ vaccination resulted in a marked increase of the M1/M2 TAMs ratio (3.0 vs. 3.9) within B16‐F10 tumors (Figure 6J). Furthermore, P@Rg‐P_50_ vaccinated B16‐F10 tumors exhibited a highly enriched CD8^+^ T cell population (45.4% vs. 54.9%) and an elevated CD8^+^/CD4^+^ T cell ratio (1.5 vs. 1.0) compared to those treated with P@Rg, indicating enhanced immune activation and improved surveillance (Figure 6K–M). Several pro‐inflammatory cytokines (IL‐1β, IFN‐γ, TNF‐α, and IL‐12) were also found to be significantly elevated in the TME of P@Rg‐P_50_ vaccinated B16‐F10 tumors, indicating an activated state of the immune system to impede tumor growth (Figure 6N–Q). Collectively, the favorable immune cells composition in tumor indicate that the P@Rg‐P_50_ vaccine can effectively enhance the activation of the host's immune cells, enabling them to rapidly recognize and precisely target tumor cells. Importantly, the absence of weight loss in the mice further underscores the safety of the treatment (Figure S49).

To further validate whether the observed long‐term tumor protection was associated with immunological memory, vaccinated mice were subjected to a secondary tumor rechallenge. Upon rechallenge, mice receiving P@MS‐P_50_ exhibited markedly delayed tumor regrowth compared with the control groups, indicating the establishment of functional antitumor immune memory (Figure S50). To further characterize this response, memory T‐cell subsets were analyzed by flow cytometry using CD44, CD62L, and CD127 markers. The treated mice displayed increased frequencies of CD44^+^ CD62L^+^ central memory T cells and CD44^+^ CD62L^+^ effector memory T cells, together with an elevated CD44^+^ CD127^+^ memory precursor population, suggesting enhanced memory formation and long‐term recall potential (Figure S51). These data collectively demonstrate that P@MS‐P_50_ not only suppresses primary tumor growth but also promotes durable antitumor immunological memory.

## 3 Conclusion

This study engineered Chol‐PItEG_m_‐modified PEAMP (P‐P_m_) nanovesicles with tunable rigidity (0.69 ± 0.05 to 4.65 ± 0.17 GPa) by precisely regulating the chain length of Chol‐PItEG_m_. As delivery vehicles, P‐P_m_ nanovesicles could efficiently co‐encapsulate R848 and gp100 peptide antigen to form a melanoma vaccine (P@Rg‐P_m_). Our investigations demonstrated that the P‐P_m_ nanovesicles significantly enhanced contact and subsequent internalization by TAMs in a rigidity‐dependent manner. More importantly, mechanical cues triggered by the P‐P_m_ nanovesicles drove TAMs to undergo repolarization from an M2‐like state toward an M1 phenotype and enhanced their antigen‐processing functions, thereby providing mechanically induced adjuvanticity. This process relied on activation of the Piezo1‐dependent mechanotransduction cascade and the YAP/TAZ coactivators. In both therapeutic and prophylactic models of B16‐F10 tumor, P@Rg‐P_50_ nanovesicles successfully reprogrammed M2‐like TAMs, activated CD8^+^ T cells, and reversed immunosuppressive TME, contributing to effective tumor growth inhibition. Notably, in the prophylactic model, P@Rg‐P_50_ significantly promoted memory T cell formation, highlighting its role in long‐term immune protection. These findings collectively highlight the mechanically induced adjuvanticity of P‐P_m_ nanovesicles, which effectively potentiate antitumor immunity through TAMs reprogramming, offering an innovative strategy for cancer vaccine design.

While P@Rg‐P_50_ nanovaccine demonstrates great potentials for cancer immunotherapy, several issues and challenges must be addressed before clinical translation. First, considering mechanosensation is often a synergistic process involving multiple sensors besides Piezo1 ion channel, future investigations would be extended to fully elaborate the individual and collective contributions of other mechanosensors, such as integrins [50], transient receptor potential channels [54], and caveolae [35, 55], in the rigidity‐dependent adjuvanticity of P‐P_m_ nanovesicles. Moreover, mechanistic studies in more depth on crosstalk between YAP/TAZ and NF‐κB/MyD88 signaling need to be explored [56, 57]. Second, in this study, the rigid nanovesicles are engineered with esterase‐sensitive linkages, ensuring their intracellular degradation shortly after providing the initial mechanical stimulus to macrophages. This transient and intermittent delivery of mechanical signals may mitigate the risk of long‐term compensatory matrix stiffening typically associated with persistent physical implants. Nevertheless, it is necessary to examine the possible occurrence of “mechanical resistance” or desensitization during long‐term administration and prolonged mechanical stimulation with the aim of confirming the durability of mechanotherapy. Third, although significant antitumor efficacy was observed in young female C57BL/6 mice, future studies would validate this strategy across broader demographic settings, as the clinical cancer population comprises patients of different ages and genders with diverse age‐related immunosenescence and sex‐hormone‐dependent immune modulation. Finally, in view of the product quality for clinical transformation, consistent mechanical control in scalable production and across batches is essential, especially in the performance of Young's modulus, stiffness, and bending modulus, which is based on the precise construct of rigid polymeric carriers. Moreover, a standardized protocol to fabricate antigen‐loaded rigid nanovesicles is also critical to achieve significant clinical outcome.

## 4 Experimental Section

### 4.1 Materials

Hexachlorocyclotriphosphazene, methoxy‐poly(ethylene glycol)2000 (mPEG2000), 2‐aminoethyl methacrylate (AEMA) hydrochloride, and trans‐dichlorobis(triphenyl‐phosphine) palladium (II) were purchased from Sigma–Aldrich Chemical Reagent Co., Ltd. 4‐Aminobenzoic acid, formic acid, acetic anhydride, tetraethylene glycol monomethyl ether, trimethylamine, triphosgene, 3‐bromopropyne, 3‐butyn‐1‐ol, cholesterol, cuprous chloride, 1‐(3‐dimethylaminopropyl)‐3‐ethylcarbodiimide (EDC), 4‐dimethylaminopyridine (DMAP), and N‐hydroxy succinimide (NHS) were purchased from Aladdin Reagents Co., Ltd. R848 was purchased from TOMUMS life science Co., Ltd. gp100 peptides and FITC modified‐gp100 peptides were synthesized by Sangon Biotech (Shanghai) Co., Ltd. All compounds, reagents or solvents were used without further purification. Roswell Park Memorial Institute (RPMI) 1640 Medium, Dulbecco's Modified Eagle's Medium (DMEM) and fetal bovine serum (FBS) were purchased from Gibco Laboratories. Interleukin‐4 (IL‐4, cat.214‐14), Interleukin‐2 (IL‐2, cat.212‐12), macrophage colony‐stimulating factor (M‐CSF, cat.315‐02) and γ‐interferon (IFN‐γ, cat.315‐05) were purchased from PeproTech, Inc. Lipolyaccharide (LPS, cat. S11060) was purchased from Shanghai Yuanye Bio‐Technology Co., Ltd. Annexin V‐FITC Apoptosis Detection Kit was purchased from Yeasen Biotechnology (shanghai) Co., Ltd. CCK‐8 was purchased from Yi‐He Biotech Co., Ltd. CFSE, Hoechst 33342, ActinGreen 555 ReadyProbes, Flou‐4‐AM, Reverse transcription kit, and PowerUp SYBR Green Master Mix were purchased from Thermo Fisher Scientific. Verteporfin and GsMTx4 was purchased from MedChemExpress LLC. Fixable Viability Dye eFluor 450 was purchased from eBioscience Co., Ltd. All ELISA kits were purchased from Abcam.

### 4.2 Cell Lines and Animals

RAW264.7, B16‐F10, CT‐26, Hepa1‐6, A549, 4T1, L929, HUVEC, C166 cells were obtained from the Type Culture Collection of the Chinese Academy of Sciences and the China Center for Type Culture Collection. BMDMs were harvested from the bone marrow of female C57BL/6 mice (6–7 weeks) and stimulated with M‐CSF (50 ng mL^−1^) for a duration of 7 days, with culture medium refreshed on the third day [14, 58]. The RAW264.7, BMDMs, Hepa1‐6 and C166 cells were cultured in DMEM high glucose medium containing 10% FBS and 1% penicillin‐streptomycin; the B16‐F10, CT‐26, 4T1, L929 and HUVEC cells were cultivated in RPMI 1640 medium containing 10% FBS and 1% penicillin‐streptomycin. All cells were kept in an incubator at 37°C with 5% CO_2_.

M1‐like macrophages were generated by exposing RAW264.7 cells or BMDMs (M0 state) to a combination of LPS (20 ng mL^−1^) and IFN‐γ (20 ng mL^−1^) for a duration of 24 h. M2‐like macrophages were induced by treating RAW264.7 cells or BMDMs (M0 state) with IL‐4 (20 ng mL^−1^) for 24 h [58].

Female C57BL/6 mice (18–20 g, 6–7 weeks) were offered by Shanghai SLAC Laboratory Animal Co., Ltd. All animals were maintained under specific pathogen‐free conditions with optimal temperature, sufficient food, and water. All animal experiments were performed under the guidance and approved by the Institutional Animal Care and Use Committee, Zhejiang Center of Laboratory Animals (approval No. ZJCLA‐IACUC‐20011101; No. ZJCLA‐IACUC‐20011521). All efforts were made to minimize animal suffering and to use the minimum number of animals necessary to obtain statistically significant data.

### 4.3 Synthesis and Characterization of PEAMP and Chol‐PItEG_m_

PEAMP was synthesized through a sequential substitution reaction of mPEG_2000_‐NH_2_/AEMA on the poly(dichlorophosphazene) backbone. Chol‐PItEG_m_ was synthesized by cholesterol‐palladium(II) (Chol‐Pd(II)) complex initiated living polymerization of 4‐isocyanobenzoic acid tetraethylene glycol monomethyl ether ester (ItEG) monomer. The chemical structures of PEAMP and Chol‐PItEG_m_ was characterized by ^1^H NMR spectrometer and FT‐IR spectrometer. The molecular weight distribution of Chol‐PItEG_m_ was determined by GPC. Detailed synthesis methods and characterization information is provided in Supporting Information.

### 4.4 Preparation of P@Rg‐P_m_

Typically, PEAMP (10 mg) and R848 (1 mg) were dissolved in 0.1 mL N, N‐dimethylformamide (DMF) and stirred for thoroughly mixing. Subsequently, an equal volume of gp100 peptides (5 mg mL^−1^) aqueous solution was added dropwise to the mixture under stirring. The resulting mixture was then dialyzed against distilled water to remove free R848/gp100 peptides and organic solvent with frequent water changes for 4–6 h. PEAMP nanovesicles co‐loaded with R848/gp100 peptides were obtained (termed as P@Rg). Next, P@Rg was mixed with Chol‐PItEG_m_ (Chol‐PItEG_20_, Chol‐PItEG_30_, or Chol‐PItEG_50_, respectively) at a final concentration of 1 mm. The resulting solution was incubated in a thermostatic shaking incubator at 37°C for 1 h and then dialyzed against distilled water. Chol‐PItEG_m_‐decorated P@Rg were obtained (termed as P@Rg‐P_m_).

Blank PEAMP nanovesicles were prepared using the same method as described above, with the exception of the addition of R848/gp100 peptides and the decoration of Chol‐PItEG_m_. R848‐loaded PEAMP nanovesicles (denoted as P@R) and gp100‐loaded PEAMP Nanovesicles (denoted as P@g) were also prepared using the identical method, incorporating only R848 or gp100 peptides, respectively. P‐P_m_ were fabricated by mixing blank PEAMP nanovesicles and Chol‐PItEG_m_ (Chol‐PItEG_20_/Chol‐PItEG_30_/Chol‐PItEG_50_) in a thermostatic shaking incubator at 37°C for 1 h and then dialyzed against distilled water. The full names and abbreviations of various nano‐agents were listed in Table S6.

The prepared P@Rg‐P_m_ was disassembled in DMF solvent and centrifuged to collect the supernatant for EE and LC measurements. Specifically, R848 loaded in P@Rg‐P_m_ was measured by using UV–visible absorption spectrophotometry (J‐1500, JASCO, Japan) at 290 nm. gp100 peptides was labeled with FITC for fluorescence visualization and measured using a fluorescence spectrophotometer. The EE and LC of P@Rg‐P_m_ were determined as follow formulas:

(1) $$\mathrm{EE}\;\left(\%\right)=\frac{\mathrm{Mass}\;\mathrm{of}\;\mathrm{loaded}\;R848/\mathrm{gp}\;100}{\mathrm{Mass}\;\mathrm{of}\;\mathrm{added}\;R848/\mathrm{gp}\;100}\times 100\%$$

(2) $$\mathrm{LC}\;\left(\%\right)=\frac{\mathrm{Mass}\;\mathrm{of}\;\mathrm{loaded}\;R848/\mathrm{gp}\;100}{\mathrm{Mass}\;\mathrm{of}\;P@\mathrm{Rg}-P_{m}}\times 100\%$$

### 4.5 Contact Angle Measurement

The static contact angles of Chol‐PItEG_20_, Chol‐PItEG_30_, and Chol‐PItEG_50_ with deionized water were measured using a Video Optical Contact Angle measuring instrument (OCA 20, DataPhysics). Thin polymer films were prepared by coating 30 mg/mL chloroform solutions of each sample onto pre‐cleaned glass substrates, followed by vacuum drying to eliminate residual solvent. A 2 µL droplet of deionized water was dispensed onto the polymer‐coated surfaces under controlled conditions. Droplet images were captured within 5 s of deposition to minimize evaporation effects, and contact angles were calculated using instrument‐integrated software.

### 4.6 Confocal Microscopy for Structural Characterization of PEAMP Nanovesicles

Typically, PEAMP and Nile Red were dissolved in 0.1 mL DMF and stirred for thoroughly mixing. Subsequently, an equal volume of gp100‐FITC peptides aqueous solution was added dropwise to the mixture under stirring. The resulting mixture was then dialyzed against distilled water to remove free Nile/gp100‐FITC peptides and organic solvent with frequent water changes for 4–6 h. Following dialysis, a 10 µL aliquot of the purified nanoformulation was deposited onto a pre‐cleaned glass slide and air‐dried at room temperature in a light‐protected environment to prevent fluorophore degradation. The dried sample was imaged using a Nikon N‐STORM/A1R super‐resolution confocal system. Fluorescence signals from FITC‐labeled gp100 peptides and Nile Red were acquired in sequential scanning mode to eliminate spectral cross‐talk.

### 4.7 In Vitro Cumulative Release of P@Rg‐P_50_

A phosphate buffered saline (PBS) at pH levels of 7.4 and 5.5 was prepared to serve as the release medium. P@Rg‐P_50_ was loaded into a dialysis bag (3500 Da MWCO) and submerged into the PBS. The release medium, containing P@Rg‐P_50_, was then placed in a thermostatic shaker set at 37°C to ensure temperature maintenance and homogeneity. Samples were periodically collected from the release medium, and the percentage release of R848 and gp100 peptides from P@Rg‐P_50_ was determined through concentration measurements of R848 and gp100 peptides. Finally, a release curve was plotted to illustrate the cumulative release behavior of R848 and gp100 peptides in P@Rg‐P_50_ over time.

### 4.8 Atomic Force Microscopy

A small volume of PEAMP/P‐P_20_/P‐P_30_/P‐P_50_ in PBS or 10% SCM was deposited onto the surface of a clean silicon wafer (1 cm × 1 cm) and allow it to air‐dry to form a film. Then, the sample was washed with deionized water for three times to remove excess nanoparticles and dried in the air. Subsequently, AFM (Dimension ICON, Bruker, Germany) was employed in Peakforce QNM mode with a scan rate of 0.5 Hz to characterize the size and mechanical properties of PEAMP/P‐P_20_/P‐P_30_/P‐P_50_. The RTESPA‐300 probe with a spring constant of 37.3 N m^−1^ and a tip radius of 8 nm was used in this experiment. All image data were processed and analyzed by Nanoscope Analysis software (Bruker).

### 4.9 Reprogramming Effects of P@Rg‐P_m_ on M2‐Like Macrophages

M2‐like RAW264.7/BMDMs were seeded in 12‐well plates and were incubated in an incubator at 37°C with 5% CO_2_ for 24 h. Following incubation, the cells were exposed to PEAMP, P@R, P@g, P@Rg, P@Rg‐P_20_, P@Rg‐P_30_, or P@Rg‐P_50_ at equivalent concentrations of 0.2 mg mL^−1^ PEAMP, 1 µg mL^−1^ R848, 1 µg mL^−1^ gp100 peptides, and 0.02 mm Chol‐PItEG_m_ for another 24 h. Then, macrophages were harvested, and the supernatant was collected for cytokine detection using an ELISA kit. The collected cells were wash with PBS for removing excess nano‐agents and then stained with fluorescently labeled antibodies against CD80, CD86, CD206, and MHC II. Flow cytometry (Beckman Coulter, Cytoflex, USA) was employed for the detection of surfac marker expression.

### 4.10 Quantitative Real‐Time Polymerase Chain Reaction (qRT‐PCR)

qRT‐PCR was employed to evaluate the expression levels of inflammatory genes in M2‐like macrophages. Briefly, M2‐like RAW264.7 cells were seeded in cell culture plates and allowed to adhere overnight. The cells were treated with PEAMP, P@R, P@g, P@Rg, P@Rg‐P_20_, P@Rg‐P_30_, or P@Rg‐P_50_ at equivalent concentrations of 0.2 mg mL^−1^ PEAMP, 1 µg mL^−1^ R848, 1 µg mL^−1^ gp100 peptides, and 0.02 mm Chol‐PItEG_m_ for a duration of 24 h. Following the treatment, the cells were collected and subjected to total RNA extraction by using Trizol reagent. Subsequently, the extracted RNA was quantified using NanoDrop (NanoDrop One, Thermo Fisher Scientific, USA), and a reverse transcription kit was used to reversing transcription of RNA to cDNA. Finally, qRT‐PCR reaction mixture containing cDNA, gene‐specific primers, and PowerUp SYBR Green Master Mix was prepared. The mixtures underwent amplification and detection using a qRT‐PCR detection system (CFX96, BioRad, USA) with optimized cycling conditions. Data analysis was performed by using the comparative Ct method (ΔΔCt). The primers information was provided in Table S7.

### 4.11 Phagocytosis of B16‐F10 Cells by P@Rg‐P_m_‐Reeducated M2‐Like Macrophages In Vitro

B16‐F10 cells were stained with CFSE (1 µM) as per the manufacturer's instructions. M2‐like RAW264.7 cells were pre‐treated with PEAMP, P@R, P@g, P@Rg, P@Rg‐P_20_, P@Rg‐P_30_, or P@Rg‐P_50_ at equivalent concentrations of 0.2 mg mL^−1^ PEAMP, 1 µg mL^−1^ R848, 1 µg mL^−1^ gp100 peptides, and 0.02 mm Chol‐PItEG_m_ for 24 h. Subsequently, the reeducated M2‐like macrophages were stained with DiI (5 µm) and co‐cultured with CFSE‐labeled B16‐F10 cells at a ratio of 1:1 for 24 h. Finally, the cells were harvested and analyzed by a flow cytometer (Beckman Coulter, Cytoflex, USA). The phagocytosis rate of B16‐F10 cell by reeducated M2‐like macrophages was calculated as follow formulas:

(3) $$\mathrm{Phagocytosis}\;\left(\%\right)=\frac{\mathrm{CFS}E^{+}\mathrm{Di}I^{+}\;\mathrm{Cells}}{\mathrm{Di}I^{+}\;\mathrm{Cells}\;}\times 100\%$$

### 4.12 The Proliferation, Activation of T Cells by P@Rg‐P_m_‐Reeducated M2‐Like Macrophages and Their Cytotoxicity Against Tumor Cells

Naïve CD8^+^ T cells were isolated from healthy C57BL/6 mice spleens by using the MojoSort Mouse CD8 T Cell isolation Kit according to the vendors’ protocol. The isolated cells were then cultured in RPMI 1640 medium containing 10% FBS and 1% penicillin‐streptomycin, supplemented with IL‐2 (20 ng mL^−1^) for subsequent experiments. M2‐like BMDMs were treated with PEAMP, P@R, P@g, P@Rg, P@Rg‐P_20_, P@Rg‐P_30_, or P@Rg‐P_50_ at equivalent concentrations of 0.2 mg mL^−1^ PEAMP, 1 µg mL^−1^ R848, 1 µg mL^−1^ gp100 peptides, and 0.02 mm Chol‐PItEG_m_ for 24 h. To evaluate T cell proliferation, CD8^+^ T cells were labeled with CFSE (1 µm) and co‐cultured with the pretreated M2‐like BMDMs at a ratio of 10:1 for 72 h. Following co‐culture, CFSE‐labeled CD8^+^ T cells were collected and then stained with anti‐CD8a‐APC for flow cytometric analysis (Beckman Coulter, Cytoflex, USA). CFSE dilution was taken as an indicator for evaluating the proliferation of CD8^+^ T cells. For assessing the activation of CD8^+^ T cells, the supernatant was collected for detecting the cytokine IFN‐γ and TNF‐α by ELISA kit according to the manufacturer's protocols. To evaluate the cytotoxic activity of antigen‐specific CD8^+^ T cells against B16‐F10 cells, the above collected CD8^+^ T cells were co‐cultured with B16‐F10 cells at a ratio of 10:1 for 24 h. Following the co‐culture, B16‐F10 cells were collected and subjected to apoptosis analysis using the Annexin V‐FITC apoptosis detection Kit according to the manufacturer's instructions.

### 4.13 Cytotoxicity of P@Rg‐P_m_‐Reeducated M2‐Like Macrophages against Tumor Cells

M2‐like macrophages were seeded in 12‐well plates and treated with PEAMP, P@R, P@g, P@Rg, P@Rg‐P_20_, P@Rg‐P_30_, or P@Rg‐P_50_ at equivalent concentrations of 0.2 mg mL^−1^ PEAMP, 1 µg mL^−1^ R848, 1 µg mL^−1^ gp100 peptides, and 0.02 mm Chol‐PItEG_m_ for 24 h to induce M1 phenotype. Subsequently, the supernatant was collected and centrifugated for removing cell debris. Then, the collected supernatant was then transferred to tumor cells (B16‐F10, CT‐26, Hepa1‐6, A549, 4T1 cells) pre‐seeded in 96‐well plates and incubated for 24 h. After the incubation, the viability of tumor cells was assessed by CCK‐8 assay according to the manufacturer's instructions.

### 4.14 Proliferation of B16‐F10 Cells Impacted by P@Rg‐P_m_‐Reeducated M2‐Like Macrophages

Transwell co‐culture system was utilized to assess the impact of P@Rg‐P_m_‐reeducated M2‐like macrophages on B16‐F10 cell proliferation. Briefly, M2‐like RAW264.7 cells were plated in the lower‐chamber of Transwell and exposed to PEAMP, P@R, P@g, P@Rg, P@Rg‐P_20_, P@Rg‐P_30_, or P@Rg‐P_50_ at equivalent concentrations of 0.2 mg mL^−1^ PEAMP, 1 µg mL^−1^ R848, 1 µg mL^−1^ gp100 peptides, and 0.02 mm Chol‐PItEG_m_ to induce M1 phenotype. Meanwhile, B16‐F10 cells were labeled with CFSE (1 µm) and were seeded in the upper chamber of the Transwell. After 24 h of co‐incubation, the CFSE‐labeled B16‐F10 cells in the upper chamber were collected and subjected to flow cytometric analysis (Beckman Coulter, Cytoflex, USA). CFSE dilution was taken as an indicator for evaluating the proliferation of B16‐F10 cells.

### 4.15 Cellular Uptake Assay

The cellular uptake efficiency of P‐P_m_ by RAW264.7 cells was examined by a CLSM and flow cytometry. Briefly, M2‐like RAW264.7 cells were seeded in cell slides and incubated in an incubator at 37°C with 5% CO_2_ for 24 h. After cell adhesion, the cells were treated with PEAMP, P‐P_20_, P‐P_30_, or P‐P_50_ at equivalent concentrations of 0.2 mg mL^−1^ PEAMP and 0.02 mm Chol‐PItEG_m_ for different time intervals. Fluorescent dye Nile red was loaded in PEAMP, P‐P_20_, P‐P_30_, or P‐P_50_ for tracking and detecting. Then, the cells are washed with PBS for three times and fixed with 4% paraformaldehyde for 15 min. Cell nuclei were stained with Hoechst 33342. Cell imaging was performed using a CLSM (N‐STORM/A1R, Nikon, Japan) equipped with a 60 × objective. For the flow cytometric analysis, M2‐like macrophages were seeded in 12‐well plates and stimulated with PEAMP, P‐P_20_, P‐P_30_, or P‐P_50_ at equivalent concentrations of 0.2 mg mL^−1^ PEAMP and 0.02 mm Chol‐PItEG_m_ for the same time intervals. Then, the cells were harvested by using trypsin digestion and washed with PBS for three times to remove the excess nano‐agents. Finally, cells were subjected to flow cytometry analysis (Beckman Coulter, Cytoflex, USA).

### 4.16 Cellular Uptake Mechanisms

RAW264.7 cells were seeded in 12‐well plates and pretreated for 2 h with the following inhibitors: Chlorpromazine (10 µg/mL) to block clathrin‐mediated endocytosis, Genistein (100 µg/mL) to inhibit caveolae‐mediated endocytosis, Nocodazole (100 ng/mL) to disrupt microtubule‐dependent trafficking, Amiloride (40 µg/mL) to suppress macropinocytosis. Subsequently, RAW264.7 cells were exposed to PEAMP, P‐P_20_, P‐P_30_, or P‐P_50_ loaded with fluorescent dye Nile red for another 4 h and subjected to flow cytometric analysis.

### 4.17 Isothermal Titration Calorimetry Assay

The thermodynamic interactions between P‐P_m_ (P‐P_20_/P‐P_30_/P‐P_50_) and RAW264.7 cells were measured using an ITC (MicroCal VP‐ITC, Germany) equipped with a 1.43 mL sample cell and a 280 µL injection syringe. Briefly, P‐P_m_ were suspended in saline to prepare a stock solution, and RAW264.7 cells were harvested and resuspend in saline. The isothermal titration calorimeter was equilibrated to 37°C. Subsequently, the P‐P_m_ (10 mg mL^−1^) suspension loaded in the titrant syringe was injected into the sample cell containing RAW264.7 cells, with a continuous stirring speed rate of 307 rpm. The titration procedure was set with an initial injection volume of 5 µL, followed by subsequent injections of 10 µL each. Every injection was spaced at intervals of 120 s, and 27 injections were administered during the titration process in total. The generated heat during each injection was quantified by integrating the area under each heat burst curve using Origin 7.0 software. Heat changes were record associated with the binding of RAW264.7 cells to P‐P_m_ throughout the titration process. The evaluation of binding affinity was determined by measuring the∆P between the maximum heat signal and the equilibrium state.

### 4.18 Forces Measurement in the Process of Macrophage Interaction with P‐P_m_

A single molecular optical tweezer system (C‐trap Dymo400, Lumicks, Netherlands) was used for the generated forces measurement between PEAMP/P‐P_20_/P‐P_30_/P‐P_50_ and the cell membrane. Briefly, for cell preparation, Raw264.7 cells were seeded in glass slides (8 × 10^3^ cells per slide) and were incubated in an incubator at 37°C with 5% CO_2_ for cell adhesion. Then, the cells were fixed by 4% paraformaldehyde prior to testing. For beads preparation, mPEG_2000_/HO‐PItEG_20_/HO‐PItEG_30_/HO‐PItEG_50_ were modified in carboxyl latex beads (4% w/v, 2 µm; Invitrogen, C37278) via esterification reaction (termed as mPEG_2000_‐beads/P_20_‐beads/P_30_‐beads/P_50_‐beads). The resulting stock solution was diluted with saline for later use. Subsequently, the prepared mPEG_2000_‐beads//P_20_‐beads/P_30_‐beads/P_50_‐beads (20 µL) was added into a slide with a flat bottom groove (slide size: 25 mm × 79 mm × 0.9 mm; groove size: 15 mm × 15 mm ×0.1 mm, volume: ∼22 µL). The prepared glass slide with attached RAW264.7 cells was sealed onto the groove to form a micro‐chamber, ensuring full contact between the RAW264.7 cells and the beads within the groove. Afterward, the micro‐chamber was positioned horizontally on the nano‐stage and aligned perpendicular to the oil immersion condenser (60 ×, Numerical Aperture 1.4, Leica) from above and the water immersion objective (60 ×, Numerical Aperture 1.2, Nikon) from below. The infrared (IR) laser beams (1064 nm) were directed in the micro‐chamber by the objective, and the condenser effectively captured photons from the IR trapping beams and projected them onto position‐sensitive detectors for precise measurement of trap forces. The IR trapping power was set at 5% overall power and 100% trapping laser for Trap 1 split, which is about is about ∼0.2 pN nm^−1^ in trap stiffness for a 2 µm bead (bead corner frequency ∼1950 Hz). Subsequently, the bead was captured by Trap 1 and was directed toward the target cell at a controlled speed of 1 µm s^−1^. Upon contacting the cell, the bead remains stationary for 4 s before retreating. This advancement and retreat motion is repeated for five cycles. The generated force was recorded, and the cells were optically imaged by a condenser with a 13% LED bright‐field source.

### 4.19 Cell Bounding Avidity Assay

The cell bounding avidity assay was measured z‐Movi Cell Avidity Analyzer (LUMICKS B.V.). Briefly, P‐P_m_ were pre‐coated on the chips and ensure even distribution. RAW264.7 cells were then added to the P‐P_m_ coated chip and incubated for 2 min to allow cell‐nanoparticle interaction. Acoustic force was gradually increased, and the binding cells were recorded during the assay.

### 4.20 Immunofluorescence Assay

M2‐like RAW264.7 cells were incubated with PEAMP, P‐P_20_, P‐P_30_, or P‐P_50_ at equivalent concentrations of 0.4 mg mL^−1^ PEAMP, 0.04 mm Chol‐PItEG_m_ in cell slides for 4 h. Afterward, the cells were washed with PBS for three times and fixed with 4% paraformaldehyde for 15 min. The cells were then permeabilized with 0.1% Triton X‐100 in PBS for 10 min, and blocked with 5% BSA in PBS for 1 h at room temperature. Incubate the cells with primary antibody against YAP1 overnight at 4°C. Following the incubation, the cells were stained with F‐actin (ActinGreen 555 ReadyProbes) for 15 min and Hoechst 33342 for 10 min. The cells was washed with PBS thoroughly to remove excess dye. Cell imaging was performed using a CLSM (N‐STORM/A1R, Nikon, Japan) equipped with a 60 × objective.

### 4.21 Western Blot Analysis

Macrophages were exposed to different nano‐agents for a certain duration. After treatment, the cells were lysed on ice for 30 min using RIPA lysis buffer supplemented with 1% protease inhibitor. The lysates were collected via centrifugation at 15 000 g for 30 min and quantified using the BCA assay (Beyotime, cat. P0009). The proteins obtained were then mixed with SDS‐PAGE sample loading buffer and heated to 95°C for 5 min to achieve denaturation. Then, the protein was loaded onto 10% bis‐tris gels and electrophoresed at 80 V for 20 min followed by 110 V for 60 min. The proteins were transferred to a PVDF membrane at 250 mA on ice for 100 min and was subsequently blocked with blocking buffer for 1 h at room temperature. Afterward, the membrane was incubated with primary antibodies at 4°C overnight and was washed with Tris‐buffered saline with Tween 20 (TBST) for 30 min. Finally, the membrane was incubated with fluorescein‐labeled secondary antibody for 1 h at room temperature and washed with TBST for 30 min. The blots were visualized with a C328 Odyssey CLx Infrared Imaging System (LI‐COR, USA). The antibodies information was provided in Table S8.

### 4.22 Calcium Inflow Detection

M2‐like RAW264.7 cells were seeded onto confocal dishes and incubated overnight for cell adhesive. Prior to experimentation, the cells were washed with Hank's Balanced Salt Solution (HBSS) without calcium and magnesium for removing residual calcium. Afterward, a Fluo‐4‐AM loading solution (3 µM) was prepared following the manufacturer's protocols and incubated with macrophages in darkness at 37°C for 30 min. Subsequently, the cells were washed with HBSS and incubated in HBSS for another 30 min for de‐esterification of Flou‐4‐AM. For the GsMTx4‐treated samples, GsMTx4 (3 µm) in HBSS was added to the macrophages and incubated for 30 min. Subsequently, the cells were placed on the stage of a confocal microscope (IX81‐FV1000, Olympus, Japan) equipped with a live‐cell workstation. The imaging chamber was kept at a constant temperature of 37°C and maintained with 5% CO_2_ to support optimal cellular activity. The cells were treated with PBS, Yoda1 (5 µm), PEAMP, P‐P_20_, P‐P_30_, and P‐P_50_ at equivalent concentrations of 0.2 mg mL^−1^ PEAMP, 0.02 mm Chol‐PItEG_m_ in DMEM complete culture medium (contain calcium). Real‐time imaging was conducted for capturing images every 15 s for a 10 min period. Post‐acquisition, the obtained images were analyzed using Image J software to quantify alterations in Fluo‐4 fluorescence intensity. The real‐time fluorescence value is denoted as F, with the initial fluorescence value represented as F_0_. ΔF, representing the change in fluorescence, was calculated as (F—F_0_) and is indicative of the calcium influx [59]. For flow cytometric analysis, the experimental procedure was consistent with the aforementioned steps, with the exception that following treatment with Fluo‐4‐AM or GsMTx4, the cells were exposed to nanoparticles for 1 h and subsequently collected for flow cytometry analysis.

### 4.23 In Vivo Antitumor Activity of P@Rg‐P_50_ in B16‐F10 Tumor‐Bearing Mice

For therapeutic studies, a subcutaneous B16‐F10 tumor model was first established. Briefly, the right hind legs of C57BL/6 mice were depilated and injected with B16‐F10 cells (5 × 10^5^ cells per mouse). When tumor volumes grow to ∼ 50 mm^3^, mice were randomly divided into six groups (*n* = 6) and were intravenously injected with saline, PEAMP, P‐P_50_, free R848+gp100 peptides (donated as Free Rg), P@Rg, or P@Rg‐P_50_ at equivalent concentrations of 50 mg kg^−1^ PEAMP, 2.5 mg kg^−1^ R848, 5 µg gp100 peptides, and 100 mg kg^−1^ Chol‐PItEG_m_. Treatments were administered at three‐day intervals for a total of five doses. The tumor volume and body weight of mice were recorded every three days. The tumor volume (V) was calculated as follow formula: V = L × W^2^ × 0.5, where L represents length and W represents width. At the end of the treatment, the mice were sacrificed, and the tumor and major organs were collected.

For prophylaxis studies, C57BL/6 mice were randomly divided into six groups and immunized with saline, PEAMP, P‐P_50_, Free Rg, P@Rg, or P@Rg‐P_50_ at equivalent concentrations of 50 mg kg^−1^ PEAMP, 2.5 mg kg^−1^ R848, 5 µg gp100 peptides, and 100 mg kg^−1^ Chol‐PItEG_m_. Treatments were administered at three‐day intervals for a total of five doses. Following the immunization, vaccinated mice were challenged with B16‐F10 cells (5 × 10^5^ cells per mouse) in the right hind legs. The tumor volume and body weight were recorded every three days thorough the treatment period. The serum antibody levels in the mice were monitored by ELISA at multiple time points throughout the experiment. On day 15, mice were euthanized, and tumors along with major organs (heart, liver, spleen, lungs, and kidneys) were harvested. Tissue samples were fixed in 4% paraformaldehyde, paraffin‐embedded, and sectioned at 5 µm thickness for histopathological evaluation. H&E staining was performed to assess general tissue morphology and metastatic lesions. Immunohistochemical analysis for Ki‐67 quantified proliferating cells in tumor sections, while TUNEL assay was employed to detect apoptotic cells. Concurrently, terminal blood samples collected via retro‐orbital bleeding were analyzed for ALT, AST, BUN, and CREA levels to assess hepatic and renal functions. For the tumor rechallenge experiment, the primary tumors were surgically resected on day 16, and the mice were then subjected to secondary tumor rechallenge on day 20. Tumor recurrence was subsequently monitored, and tumors were collected at the terminal time point for memory T cell analysis.

For evaluation of in vivo nanoparticle uptake by TAMs, B16‐F10 tumor‐bearing mice were intravenously injected with free Ce6, PEAMP@Ce6, or P‐P_50_@Ce6. Briefly, PEAMP@Ce6 was prepared by encapsulating Ce6 into PEAMP nanovesicles, while P‐P_50_@Ce6 was obtained by further decorating PEAMP@Ce6 with Chol‐PItEG_50_ to generate rigid nanovesicles. After 4 h, tumors were harvested and dissociated into single‐cell suspensions. The resulting cells were stained with anti‐CD45, anti‐CD11b, and anti‐F4/80 antibodies and analyzed by flow cytometry. TAMs were identified as CD45^+^CD11b^+^F4/80^+^ cells. The intracellular uptake of Ce6‐labeled formulations by TAMs was evaluated by measuring the MFI of Ce6 within the TAMs population.

For immune cell population analysis, the tumors and spleens were homogenized in PBS containing PMSF (1 mM). The homogenates were filtered through a 40 µm cell strainer, subjected to red blood cell lysis buffer treatment, and subsequently washed with PBS to obtain single‐cell suspensions. The supernatant was collected and centrifugated for cytokine detection using an ELISA kit. The isolated single‐cell suspensions were pre‐stained with a Fixable Viability Dye eFlou 450 to distinguish live cells and subjected to fluorescently labeled antibody staining for flow cytometric analysis. Specifically, for M1/M2‐like TAMs analysis, the cells were stained with PE/Cyanine7 anti‐CD45; Alexa Fluor 700 anti‐F4/80; FITC anti‐CD11b; PE anti‐CD80; APC anti‐CD206. For T cell infiltration analysis; the cells were stained with PE/Cyanine7 anti‐CD45; PE anti‐CD3; FITC anti‐CD4; APC anti‐CD8a. For MDSCs analysis, the cells were stained with PE/Cyanine7 anti‐CD45; FITC anti‐CD11b; APC anti‐Gr‐1. For Tregs analysis, the cells were stained with PE/Cyanine7 anti‐CD45; FITC anti‐CD4; APC anti‐CD25; PE anti‐Foxp3. For memory T cell analysis, cells were stained with PE/Cyanine7 anti‐CD45, APC anti‐CD3, PE anti‐CD8, FITC anti‐CD44, and AF700 anti‐CD62L, or with PE/Cyanine7 anti‐CD45, APC anti‐CD3, PE anti‐CD8, AF700 anti‐CD44, and FITC anti‐CD127. Antibodies were utilized following the manufacturer's protocols and incubated with the cells for 30 min at room temperature. The antibodies information was provided in Table S8. Gating strategies were provided in Figures S52–S56.

### 4.24 Statistical Analysis

The data were presented as mean ± standard deviation (SD) with at least three independent experiments. Individual comparisons were analyzed using a two‐tailed, unpaired Student's t‐test. Differences among multiple groups were evaluated using one‐way analysis of variance (ANOVA). Statistical significance was defined as ^*^ *p* < 0.05, ^**^ *p* < 0.01, ^***^ *p* < 0.001, and ^****^ *p* < 0.0001, as indicated in the figure legends; n.s. denotes not significant. Statistical analysis was conducted utilizing GraphPad Prism 8.0 software (GraphPad Software, CA).

## Author Contributions

**Bangyue Luo**: methodology, data curation, investigation, Writing – original draft, formal analysis. **Liyan Qiu**: conceptualization, supervision, funding acquisition, writing – review and editing.

## Conflicts of Interest

The authors declare no conflicts of interest.

## Supporting information




**Supporting File 1**: advs76773‐sup‐0001‐SuppMat.docx.

**Supporting File 2**: advs76773‐sup‐0002‐MovieS1.mp4.

**Supporting File 3**: advs76773‐sup‐0003‐MovieS2.mp4.

**Supporting File 4**: advs76773‐sup‐0004‐MovieS3.mp4.

**Supporting File 5**: advs76773‐sup‐0005‐MovieS4.mp4.

**Supporting File 6**: advs76773‐sup‐0006‐MovieS5.mp4.

**Supporting File 7**: advs76773‐sup‐0007‐MovieS6.mp4.

**Supporting File 8**: advs76773‐sup‐0008‐MovieS7.mp4.

## Data Availability

The data that support the findings of this study are available from the corresponding author upon reasonable request.
