# Supplementary material for: Nanovesicles With Mechanically Induced Adjuvanticity for Robust Melanoma Vaccination Toward Tumor‐Associated Macrophages
Source: Adv Sci (Weinh). 2026 Jul 27;13(58):e76773. doi: 10.1002/advs.76773 (PMC13403892; doi:10.1002/advs.76773)
Supplement: Supplementary file 1 [file ADVS-13-e76773-s004.docx]

Supporting Information

**Nanovesicles with Mechanically Induced Adjuvanticity for Robust Melanoma Vaccination towards Tumor-Associated Macrophages**

Bangyue Luo, Liyan Qiu*

Ministry of Educational (MOE) Key Laboratory of Macromolecular Synthesis and Functionalization, Department of Polymer Science and Engineering, Zhejiang University, Hangzhou 310058, China.

* Corresponding author: Prof. Liyan Qiu, Tel: +86 571 87952306, Fax: +86 571 87952306, E-mail: [lyqiu@zju.edu.cn](mailto:lyqiu@zju.edu.cn)

**S1 Experimental Section**

**S2 Supplementary Figures:** Figure S1 to S56

**S3 Supplementary Tables:** Tables S1 to S8

**S4 Supplementary videos:** Videos S1 to S7

**S1 Experimental Section**

# S1.1 General methods for synthesis and characterization

All experimental procedures were conducted in a controlled environment with a desiccated atmosphere. For compounds identification, thin layer chromatography (TLC) was conducted and visualized with ultraviolet (UV) light or iodine staining. The compounds were purified using column chromatography, with inert silica gel from Sinopharm Chemical Reagent Co., Ltd. employed as the stationary phase. ^1^H nuclear magnetic resonance (^1^H NMR) spectra and ^31^P-NMR spectra of compounds were obtained by using an NMR spectrometer (Avance Ⅲ 400 or Avance Ⅲ 500, Bruker, Germany) with deuterated solvent. The ^1^H-NMR chemical shifts were calibrated to the peak of the deuterated solvent or tetramethylsilane (set as 0 ppm). The proton signal multiplicities of compounds are abbreviated as follows: s: singlet, d: doublet, t: triplet, q: quartet, m: multiplet, dd: double doublet. Fourier transform infrared (FT-IR) spectra were acquired utilizing a fourier-transform infrared spectrometer (Nicolet 6700, Thermo Fisher Scientific, USA) employing the KBr pressed method. Molecular weights and distribution were determined *via* gel permeation chromatography (GPC) (Waters 1515, Waters, USA), with tetrahydrofuran (THF) or N, N-dimethylformamide (DMF) as the eluent. The flow rate was ste as 1 mL min^-1^, and the GPC analysis was calibrated using polystyrene standards (PSt).

The size distribution/zeta potential of nanoparticles were detected by DLS (Zetasizer Nano ZS90, Malvern, UK) at 25℃. Thermal properties of the nanoparticles were evaluated using differential scanning calorimetry (DSC, TGA/DSC3+, Mettler Toledo, Switzerland). The crystalline structure of samples was investigated using X-ray diffraction (XRD, Ultima IV, Rigaku, Japan). The surface composition of samples was analyzed using X-Ray photoelectron spectroscopy (XPS, Escalab 250Xi, Thermo Fisher Scientific, USA). Transmission electron microscopy (TEM) equipped with energy dispersive X-ray spectroscopy (EDS) (HT7700 EXALENS, HITACHI, Japan) elemental microanalysis system was used to observe the morphology and analyze the elemental distribution of nanoparticles. Briefly, a small aliquot of sample solution was deposited onto a carbon-coated copper grid and air-dried for 15 min to allow the sample to adhere to the carbon film. Afterwords, digital images and element distribution of sample was obtained by exposing the sample to the electron beam for a suitable duration. The morphology of samples was analyzed using a scanning electron microscope (SEM, HITACHI S-4800). A 10 μL aliquot of the sample dispersion was deposited on a silicon substrate and air-dried at room temperature. To enhance conductivity, the substrate was sputter-coated with a thin gold layer prior to SEM observation.

# S1.2 Synthesis of poly[(methoxy-poly(ethylene glycol))(2-aminoethyl methacrylate) phosphazene]s (PEAMP)

**S1.2.1 Synthesis of mPEG_2000_-NH_2_**. mPEG_2000_ (10.0 g, 5.0 mmol, 1.0 eqv.) was dissolved in anhydrous dichloromethane (DCM, 20 mL) and mixed with DMAP (24.4 mg, 0.2 mmol, 0.2 eqv.), triethylamine (TEA) (1.0 g, 10.0 mmol, 2.0 eqv.), and 4-nitrophenyl chloroformate (5.0 g, 25.0 mmol, 5.0 eqv.) under an argon atmosphere. The reaction mixture was stirred at 0℃ for 4 h and then at 40°C for another 24 h. Subsequently, the mixture was filtered, and the resulting filtrate was concentrated by evaporation and followed by precipitated with excess amount of ice ethyl ether. Finally, the precipitate was collected by the vacuum filtration and vacuum drying. Then mPEG-NO_2_ was obtained as a white powder. The mPEG-NO_2_ (8.0 g, 3.7 mmol, 1.0 eqv.) was mixed with ethylenediamine (0.67 g, 11.1 mmol, 3.0 eqv.) and DMAP (45.2 mg, 0.37 mmol, 0.1 eqv.) in anhydrous DCM (20 mL) under an argon atmosphere. The mixture was stirred at 0℃ for 5 h and then at 40°C for another 24 h. The product was obtained by filtration, evaporation, and precipitation as the above mention process. The mPEG_2000_-NH_2_ was obtained as a yellow powder.

**S1.2.2 Synthesis of PEAMP.** PEAMP was synthesized from poly(dichlorophosphazene) by a two-step sequential nucleophilic substitution reaction. Briefly, hexachlorocyclotriphosphazene was purified *via* sublimation at 90°C, followed by ring-opening polymerization catalyzed by 0.3 wt% aluminum chloride at 250°C for 24 h. The obtained poly(dichlorophosphazene) backbone (0.5 g) was then dissolved in anhydrous toluene (8 mL) and precipitated by anhydrous petroleum ether (PE). Afterwards, the mPEG_2000_-NH_2_ (1.25 g) was dehydrated by co-boiling rectification with toluene and was dropped into the obtained poly(dichlorophosphazene) backbone in anhydrous toluene containing TEA for 24 h at 30℃. Subsequently, an excess amount of desalted 2-aminoethyl methacrylate (AEMA) with equimolar TEA in anhydrous toluene was dropped in the mixture. The reaction system was maintained at 60℃. Following the reaction for 72 h, the mixture was filtered, and the filtrate was subjected to multiple precipitations using excess amount of ice ethyl ether. Finally, the precipitate was dissolved in DMF and then dialyzed against deionized water for 72 h with frequent changes of water. The dialysate was collected and freeze-dried to obtain PEAMP as a white powder.

## S1.3 Synthesis of cholesterol-poly(4-isocyanobenzoic acid tetraethylene glycol monomethyl ether ester)m (Chol-PItEG_m_)

**S1.3.1 Synthesis of Compound (1)**. Formic acid (4.2 g, 911.3 mmol, 12.5 eqv.) and acetic anhydride (18.6 g, 182.3 mmol, 2.5 eqv.) were premixed and stirred for 1 h at room temperature. The resulting premixture was then added dropwise to a solution of 4-aminobenzoic acid (10.0 g, 72.9 mmol, 1.0 eqv.) in ethyl acetate (EA). The reaction was stirred at room temperature for 5 h. As 4-aminobenzoic acid was completely consumed, the precipitate formed, which, along with the filtrate, was collected. Subsequently, the filtrate is further precipitated with excess amount of PE. The resulting precipitate was collected by vacuum filtration. All the obtained precipitates were washed with cold PE thoroughly and dried under vacuum to obtain compound (1) as white powder.

**S1.3.2 Synthesis of Compound (2).** Compound (1) (2.8 g, 16.7 mmol, 1.2 eqv.) and tetraethylene glycol monomethyl ether (2.9 g, 13.9 mmol, 1.0 eqv.) were dissolved in DMF (80 mL), followed by addition of EDCI (4.0 g, 20.9 mmol, 1.5 eqv.) and DMAP (0.3 g, 2.8 mmol, 0.2 eqv.). The mixture was stirred at room temperature for 24 h and tracked by TLC. Subsequently, the solvent of the mixture is removed by evaporation. The residue was washed with deionized water and 10% hydrochloric acid (100 mL), saturated NaHCO_3_ (300 mL) and brine (300 mL). The organic layer was collected and dehydrated by anhydrous Na_2_SO_4_ (25 g), filtered and concentrated by evaporation. The residue was purified by column chromatography on silica gel to gain compound (2).

**S1.3.3 Synthesis of ItEG.** Compound (2) (1.0 g, 2.8 mmol, 1.0 eqv.) and TEA (0.85 g, 8.4 mmol, 3.0 eqv.) was dissolved in anhydrous DCM. The resulting mixture was stirred at 0°C for 30 min. Subsequently, the solution of triphosgene (249.3 mg, 0.84 mmol, 0.3 eqv.) in anhydrous DCM was added dropwise the reaction system. TLC was employed to track the reaction progress. After the consumption of compound (2), the mixture was collected by evaporation and purified by silica gel column chromatography to obtain ItEG.

**S1.3.4 Synthesis of Compound (3).** Cholesterol (5.0 g, 12.9 mmol, 1.0 eqv.) was dissolved in anhydrous THF (50 mL) and followed by the addition of NaH (0.37 g, 15.5 mmol, 1.2 eqv.). The reaction mixture was stirred at room temperature for 1 h. Afterwords, 3-bromopropyne (1.84 g, 15.5 mmol, 1.2 eqv.) was added dropwise and the reaction, and the reaction process was tracked by TLC. 24 h later, the solvent of the mixture is removed and the residue was extracted by DCM (20 mL). Subsequently, the residue was washed with brine (300 mL) and dehydrated by anhydrous Na_2_SO_4_ (25 g). Finally, the compound (3) was obtained by silica gel column chromatography purification.

**S1.3.5 Synthesis of Chol-Pd(II).** Compound (3) (934.4 mg, 2.2 mmol, 1.0 eqv.), trans-dichlorobis (triphenyl-phosphine) Palladium (II) (1.7 g, 2.4 mmol, 1.1 eqv.), and cuprous chloride (43.6 mg, 0.44 mmol, 0.2 eqv.) were dissolved in anhydrous DCM, followed by the addition of anhydrous diethylamine (1.6 g, 22 mmol, 10.0 eqv.). The reaction was protected from light and maintained at room temperature for 24 h. Finally, the Chol-Pd(II) complex was collected by evaporation and purified by silica gel column chromatography.

**S1.3.6 Polymerization of Chol-PItEG_m_.** Under an argon atmosphere, ItEG and Chol-Pd(II) were mixed with different initial feed ratios (**Supplementary Table 1**) in 1,2-dichloroethane and stirred at 80°C for 24 h. To remove excess monomers, the residue was precipitated and washed by ice-cold ethyl ether. The product was then collected by filtration and dried by vacuum to obtain Chol-PItEG_m_.

## S1.4 Synthesis of P_m_-beads/mPEG_2000_-beads

**S1.4.1 Synthetic of HO-Pd(Ⅱ) complex.** Under an argon atmosphere, 3-butyn-1-ol (25.0 mg, 0.36 mmol, 1.0 eqv.), trans-dichlorobis (triphenyl-phosphine) Palladium (II) (277.6 mg, 0.39 mmol, 1.1 eqv.), and cuprous chloride (7.1 mg, 0.072 mmol, 0.2 eqv.) were dissolved in anhydrous DCM, followed by the addition of anhydrous diethylamine (263.3 mg, 3.6 mmol, 10.0 eqv.). The reaction was protected from light and reacted at room temperature for 24 h. Finaly, the HO-Pd(Ⅱ) complex was isolated by silica gel column chromatography purification.

**S1.4.2 Polymerization of HO-PItEG_m_**. Under an argon atmosphere, ItEG and HO-Pd(II) were mixed with different initial feed ratios (**Supplementary Table 4**) in 1,2-dichloroethane and stirred at 80 °C for 24 h. To remove excess monomers, the residue was precipitated and washed by ice-cold ethyl ether. The product was then collected by filtration and dried by vacuum to obtain HO-PItEG_m_.

**S1.4.3 Synthesis of P_m_-beads/mPEG_2000_-beads.** HO-PItEG_m_ or mPEG_2000_ were linked to carboxyl latex beads (4% w/v, 2 μm; Invitrogen, C37278) *via* esterification reaction. Briefly, OH-PItEG_m_ or mPEG_2000_ (~70 eqv.) and carboxyl latex beads (~1 eqv.) were dissolved in deionized water, followed by the addition of EDCI. The mixture was stirred at room temperature for 24 h. Afterwords, the product was collected by centrifugation and dialysis against deionized water for removing residual reactants. Finally, HO-PItEG_m_/mPEG_2000_ modified beads (denoted as P_m_-beads/mPEG_2000_-beads) was obtained by centrifugation and store at 4 °C as stock solution for later use.

**S2 Supplementary Figures**

 **Figure S1.** Synthetic route of mPEG_2000_-NH_2_.

**Figure S2.** ^1^H NMR spectrum (400 MHz) of mPEG_2000_-NH_2_. ^1^H NMR (400 MHz, Chloroform-*d*) δ 4.22 (s, 2H), 3.82 (dd, J = 5.8, 4.1 Hz, 2H), 3.38 (s, 3H), 3.34 (d, J = 5.9 Hz, 2H), 2.94 (d, J = 5.9 Hz, 2H).

 **Figure S3.** Synthetic route of PEAMP.

**Figure S4.** ^1^H NMR spectrum (400 MHz) of PEAMP. The molar ratio of mNH_2_-PEG_2000_ to AEMA was determined to be 0.34:1.84 based on the peak areas of the chemical shifts at 3.3 and 1.80 ppm.

**Figure S5.** FT-IR spectrum of PEAMP.

The peaks at 1341 cm^-1^ and 953 cm^-1^ in the FT-IR spectrum could be ascribed to the stretching vibration of -P=N- and -P-N- (PEAMP backbone), respectively. The peaks at 2873 cm^-1^ and 1455 cm^-1^ are assigned to the stretching vibration and deformation vibration of -CH_2_- (mPEG_2000_-NH_2_), respectively. The peak at 1105 cm^-1^ results from the stretching vibration of -C-O-C- (mPEG_2000_-NH_2_). The peaks at 1718 cm^-1^ and 1660 cm^-1^ are attributed to the stretching vibration of -C=O- and -C=C- (AEMA), respectively.

**Figure S6.** Synthetic route of monomer ItEG.

**Figure S7.** ^1^H NMR spectrum (400 MHz) of Compound (1). ^1^H NMR (400 MHz, DMSO-*d*6) δ 12.76 (s, 1H), 8.34 (d, J = 2.2 Hz, 1H), 7.94-7.85 (m, 2H), 7.69 (d, J = 9.0 Hz, 2H).

**Figure S8.** ^1^H NMR spectrum (500 MHz) of Compound (2). ^1^H NMR (500 MHz, Chloroform-*d*) δ 8.01 (dd, J = 20.5, 8.5 Hz, 3H), 7.62 (d, J = 8.4 Hz, 1H), 4.45 (q, J = 5.2 Hz, 2H), 3.82 (t, J = 4.8 Hz, 2H), 3.73-3.49 (m, 12H), 3.35 (d, J = 2.1 Hz, 3H).

**Figure S9.** ^1^H NMR spectrum (400 MHz) of ItEG. ^1^H NMR (400 MHz, Chloroform-*d*) δ 8.17-8.05 (m, 2H), 7.50-7.41 (m, 2H), 4.54-4.45 (m, 2H), 3.91-3.78 (m, 2H), 3.74-3.51 (m, 12H), 3.37 (s, 3H).

**Figure S10.** Synthetic route of initiator Chol-Pd(II) complex.

**Figure S11.** ^1^H NMR spectra of (A) cholesterol, (B) Compound (3), (C) Chol-Pd(II) complex. Compound (3): ^1^H NMR (400 MHz, Chloroform-*d*) δ 5.37 (dd, J = 4.9, 2.4 Hz, 1H), 4.19 (d, J = 2.4 Hz, 2H), 3.38 (tt, J = 11.3, 4.5 Hz, 1H), 2.40 (q, J = 2.2 Hz, 1H). Chol-Pd(II) complex: ^1^H NMR (500 MHz, Chloroform-*d*) δ 5.38-5.27 (m, 1H), 4.24 (d, J = 2.0 Hz, 2H), 3.53 (tt, J = 11.4, 4.0 Hz, 1H), 1.93 (tq, J = 7.7, 3.9 Hz, 12H), 1.17 (q, J = 8.0 Hz, 18H).

**Figure S12.** Polymerization route of Chol-PItEG_m_.

**Figure S13.** FT-IR spectra of Chol-PItEG_20_, Chol-PItEG_30_, and Chol-PItEG_50_.

**Figure S14.** ^1^H NMR spectra of Chol-PItEG_20_, Chol-PItEG_30_, and Chol-PItEG_50_.

**Figure S15.** GPC chromatograms for Chol-Pd(II)-initiated polymerization of Chol-PItEG_m_ in 1,2-dichloroethane at 80°C with PSt as internal standard.

**Figure S16.** Contact angle of Chol-PItEG_20_, Chol-PItEG_30_, or Chol-PItEG_50_ with deionized water.

**Figure S17.** SEM images of P@Rg, P@Rg-P_20_, P@Rg-P_30_, P@Rg-P_50_, scale bar: 500 nm.

**Figure S18.** Elemental analysis of P@Rg-P_50_ by EDS, scale bar: 100 nm.

**Figure S19.** (A) DSC thermograms and (B) XRD diffractograms of PEAMP, R848, physical mixture of PEAMP and R848 (PEAMP + R848), and R848-loaded PEAMP (P@R). The thermal analysis of PEAMP revealed a peak at 48.38℃, while R848 showed an endothermic peak at 187.87℃. Characteristic peaks corresponding to both compounds were observed in the DSC thermogram of the physical mixture PEAMP + R848 (48.69℃ for PEAMP and 187.17℃ for R848), indicating that no chemical reaction or binding occurred upon mixing. However, only a typical peak corresponding to PEAMP (44.39℃) was observed in the DSC thermogram of P@R, suggesting the homogeneous dispersion and encapsulation of R848 within PEAMP. XRD patterns showed that PEAMP exhibited an amorphous form with no characteristic peaks, while R848 exhibited a crystalline state with characteristic peaks in the range of 10°∼35°. Physical mixtures of PEAMP and R848 in equivalent amounts maintained typical peaks similar to those of individual PEAMP and R848, indicating that simple physical mixing does not alter their original crystal structure and properties. However, upon loading into PEAMP, the crystallization peaks of R848 disappeared, with only the amorphous signal of PEAMP remaining, suggesting successful embedding of R848 in PEAMP, forming amorphous complexes.

**Figure S20.** TEM images of PEAMP nanovesicles in PBS (pH 5.5) with esterase (1,500 ng mL^-1^) after different incubation time, scale bar: 200 nm. At 0 h, PEAMP nanovesicles exhibited intact nanovesicle morphology, characterized by well-defined, spherical structures with clear boundaries. After 2 h, some nanovesicles showed blurred edges, indicating potential membrane instability or partial degradation. By 4 h, the nanovesicles showed evident fragmentation, with their edges appearing progressively blurred. By 8 h, most vesicles showed significant shrinkage, indicating extensive structural disintegration and emphasizing the profound impact of esterase treatment on vesicular integrity.

**Figure S21.** Conformational changes of mPEG_2000_ chain and Chol-PItEG_m_ chain under external forces.

**Figure S22.** (A) Topography images of PEAMP, P-P_20_, P-P_30_, and P-P_50_ from AFM scanning in 10% SCM, scale bar: 300 nm. (B to E) The size, Young’s modulus, deformation, and energy dissipation analysis of PEAMP, P-P_20_, P-P_30_, and P-P_50_ from AFM scanning in PBS or 10% SCM (n = 6 for PBS; n = 3 for SCM). n.s., not significant.

**Figure S23.** Cellular uptake efficiency of PEAMP, P-P_20_, P-P_30_, and P-P_50_ by RAW264.7 cells at 8 h.

**Figure S24.** CLSM images (scale bar: 10 μm) and the corresponding mean fluorescence intensity of RAW264.7 cells after incubation with PEAMP, P-P_20_, P-P_30_, or P-P_50_ for 2 h, 4 h, 8 h. Neil Red dye was loaded in the nano-agents for intracellular trafficking, n = 3. Data are presented as mean ± s.d. Statistical significance was determined by one-way ANOVA with Tukey’s multiple-comparisons test. **P* < 0.05, ***P* < 0.01, ****P* < 0.001, and *****P* < 0.0001; n.s., not significant.

**Figure S25.** Synthetic route of HO-Pd(Ⅱ) complex.

**Figure S26.** (A) ^1^H NMR spectrum (400 MHz) and (B) ^31^P NMR spectrum (162 MHz) of bifunctional initiator HO-Pd(Ⅱ) complex in CDCl_3_. ^1^H NMR (400 MHz, Chloroform-*d*) δ 3.60 (q, J = 6.0 Hz, 2H), 2.51 (tt, J = 6.1, 2.1 Hz, 2H), 1.92 (qq, J = 7.5, 4.3, 3.4 Hz, 12H), 1.16 (p, J = 7.9 Hz, 18H).

**Figure S27.** Polymerization route of HO-PItEG_m_.

**Figure S28.** GPC chromatograms for HO-Pd(II)-initiated polymerization of HO-PItEG_m_ in 1,2-dichloroethane at 80°C with PSt as internal standard.

**Figure S29.** Synthesis routes of P_m_-beads/mPEG_2000_-beads.

**Figure S30.** FT-IR spectra of mPEG_2000_-beads and P_m_-beads.

**Figure S31.** (A) Schematic of M2-like RAW264.7 cells harvest process. (B, C) Representative flow cytometry histograms and mean fluorescence intensity of M0-like or M2-like RAW264.7 cells, n = 3. (D) Cytokine concentration of IL-10 in the culture supernatant of M0-like or M2-like RAW264.7 cells. n = 4. Data are presented as mean ± s.d. Statistical significance was determined by a two-tailed unpaired t-test. **P* < 0.05, ***P* < 0.01, ****P* < 0.001, and *****P* < 0.0001; n.s., not significant.

**Figure S32.** Cell viability analysis of RAW264.7, HUVEC, L929, and C166 cells treated with Chol-PItEG_20_, Chol-PItEG_30_, or Chol-PItEG_50_ in different concentration by CCK-8 assay, n = 3. **P* < 0.05, ***P* < 0.01, ****P* < 0.001, and *****P* < 0.0001; n.s., not significant.

**Figure S33.** Representative flow cytometry zebra plots of (A) CD80^+^, (B) CD86^+^, and (C) MHC II^+^ cells in M2-like RAW264.7 cells reprogrammed by PEAMP, P@R, P@g, P@Rg, P@Rg-P_20_, P@Rg-P_30_, or P@Rg-P_50_ for 24 h.

**Figure S34.** (A) Schematic of BMDMs harvest process. (B) Representative flow cytometry dot plots and (C) percentage of F4/80^+^ cells in BMDMs, n = 3. Data are presented as mean ± s.d. Statistical significance was determined by a two-tailed unpaired t-test. **P* < 0.05, ***P* < 0.01, ****P* < 0.001, and *****P* < 0.0001; n.s., not significant.

**Figure S35.** Representative flow cytometry zebra plots of (A) CD80^+^, (B) CD86^+^, (C) MHC II^+^, and (D) CD206^+^ cells in M2-like BMDMs treated with PEAMP, P@R, P@g, P@Rg, P@Rg-P_20_, P@Rg-P_30_, or P@Rg-P_50_ for 24 h. **P* < 0.05, ***P* < 0.01, ****P* < 0.001, and *****P* < 0.0001; n.s., not significant.

**Figure S36.** Cytokines detection of TNF-α, IL-1β, and IL-12p70 in the culture supernatant of M2-like BMDMs treated with PEAMP, P@R, P@g, P@Rg, P@Rg-P_20_, P@Rg-P_30_, or P@Rg-P_50_ for 24 h, n = 3. **P* < 0.05, ***P* < 0.01, ****P* < 0.001, and *****P* < 0.0001; n.s., not significant.

**Figure S37.** (A) Representative flow cytometry dot plots of phagocytosis of B16-F10 cells by PEAMP, P@R, P@g, P@Rg, P@Rg-P_20_, P@Rg-P_30_, and P@Rg-P_50_ reprogrammed-M2-like RAW264.7 cells for 24 h. (B) Representative flow cytometry histograms proliferation of CD8^+^ T cell after co-cultured with PEAMP, P@R, P@g, P@Rg, P@Rg-P_20_, P@Rg-P_30_, and P@Rg-P_50_-reprogrammed-M2-like BMDMs for 72 h. (C) Representative flow cytometry dot plots of apoptosis of B16-F10 cells after co-culture with CD8^+^ T cells activated by PEAMP, P@R, P@g, P@Rg, P@Rg-P_20_, P@Rg-P_30_, and P@Rg-P_50_-reeducated M2-like BMDMs for 24 h.

 **Figure S38.** Cell viability analysis of B16-F10, CT-26, Hepa1-6, A549, and 4T1 cells treated with PEAMP, P@R, P@g, P@Rg, P@Rg-P_20_, P@Rg-P_30_, or P@Rg-P_50_ for 24 h by CCK-8, n = 6. Data are presented as mean ± s.d. Statistical significance was determined by one-way ANOVA with Tukey’s multiple-comparisons test. **P* < 0.05, ***P* < 0.01, ****P* < 0.001, and *****P* < 0.0001; n.s., not significant.

**Figure S39.** Proliferation of B16-F10 cells co-cultured with PEAMP, P@R, P@g, P@Rg, P@Rg-P_20_, P@Rg-P_30_, or P@Rg-P_50_-reeducated M2-like RAW264.7 cells based on CFSE staining, n = 3. Data are presented as mean ± s.d. Statistical significance was determined by one-way ANOVA with Tukey’s multiple-comparisons test. **P* < 0.05, ***P* < 0.01, ****P* < 0.001, and *****P* < 0.0001; n.s., not significant.

**Figure S40.** Apoptosis rate and representative flow cytometry dot plots of 4T1 cells after co-culture with CD8^+^ T cells activated by PEAMP, P@R, P@g, P@Rg, P@Rg-P_20_, P@Rg-P_30_, or P@Rg-P_50_-reeducated M2-like BMDMs for 24 h. Statistical significance was determined by one-way ANOVA with Tukey’s multiple-comparisons test. **P* < 0.05, ***P* < 0.01, ****P* < 0.001, and *****P* < 0.0001; n.s., not significant.

 **Figure S41.** (A) Relative mRNA expression of YAP1 in RAW264.7 cells exposed to PEAMP, PEAMP, P-P_20_, P-P_30_, and P-P_50_ for 4 h, n = 3. (B to D) Surface markers expression analysis of CD80, CD86, and MHC II in M2-like RAW264.7 cells reprogrammed by PEAMP, PEAMP, P-P_20_, P-P_30_, and P-P_50_ with or without Vitiporfin (0.3 μM, 1 h) pre-treatment for 24 h, n = 3. (E) Schematic illustration of YAP/TAZ activation by P-P_m_. Statistical significance was determined by one-way ANOVA with Tukey’s multiple-comparisons test. **P* < 0.05, ***P* < 0.01, ****P* < 0.001, and *****P* < 0.0001; n.s., not significant.

**Figure S42.** Representative flow cytometry histograms of Flou-4 in RAW264.7 cells treated with complete culture medium (as control), PEAMP, P-P_20_, P-P_30_, P-P_50_, and Yoda 1 (5 μM) for 2 h with or without GsMTx4 (3 μM, 0.5 h) pre-treatment.

**Figure S43.** (A) Images of tumors harvested from mice at the end of the treatment of Saline, PEAMP, P-P_50_, Free Rg, P@Rg, or P@Rg-P_50_, n = 6. (B) Body weight change of mice throughout the treatment, n = 6.

**Figure S44.** H&E staining, TUNEL staining, and Ki67 immunohistochemical staining of tumor sections from B16-F10 tumor-bearing mice treated with Saline, PEAMP, P-P_50_, Free Rg, P@Rg, or P@Rg-P_50_ in therapeutic treatment, scale bar: 50 μm.

**Figure S45.** H&E staining of major organ sections from B16-F10 tumor-bearing mice treated with Saline, PEAMP, P-P_50_, Free Rg, P@Rg, or P@Rg-P_50_ in therapeutic treatment, scale bar: 100 μm.

 **Figure S46.** Serum ALT, AST, BUN, CREA level of mice treated with Saline, PEAMP, P-P_50_, Free Rg, P@Rg, or P@Rg-P_50_ in therapeutic treatment, n = 3. Data are presented as mean ± s.d. Statistical significance was determined by one-way ANOVA with Tukey’s multiple-comparisons test. **P* < 0.05, ***P* < 0.01, ****P* < 0.001, and *****P* < 0.0001; n.s., not significant.

**Figure S47.** (A) Representative flow cytometry gating strategy for identifying TAMs in tumor single-cell suspensions isolated from B16-F10 tumors. (B) Flow cytometry histograms showing intracellular Ce6 fluorescence in TAMs after treatment with free Ce6, PEAMP@Ce6, or P-P_50_@Ce6 nanoparticles. (C) Quantification of MFI of Ce6 in TAMs, n = 5. Statistical significance was determined by one-way ANOVA with Tukey’s multiple-comparisons test. **P* < 0.05, ***P* < 0.01, ****P* < 0.001, and *****P* < 0.0001; n.s., not significant.

 **Figure S48.** Serum IgG1 and IgG2a antibody analysis of mice treated with Saline, PEAMP, P-P_50_, Free Rg, P@Rg, or P@Rg-P_50_ in prophylactic treatment. n = 3.

**Figure S49.** Body weight change of mice throughout the prophylactic treatment, n = 6.

**Figure S50.** (A) Schematic of the B16-F10 tumor rechallenge model in mice treated with Saline, PEAMP, P-P_50_, Free Rg, P@Rg, or P@Rg-P_50_. (B) Individual tumor growth curves of B16-F10 tumor-bearing mice following tumor rechallenge, n = 6. (C) Representative images of tumors harvested from mice at the end of the rechallenge experiment, n = 6.

**Figure S51.** (A) Gating strategy and representative flow cytometry plots for memory T cell subsets. (B to D) Representative flow cytometry plots and quantification of CD44^+^ CD62L^-^ or CD44^+^ CD62L^+^ memory T cells in the spleen the indicated groups, n = 6. (E, F) Representative flow cytometry plots and quantification of CD44^+^ CD127^+^ memory T cells in the spleen from the indicated groups, n = 6. **P* < 0.05, ***P* < 0.01, ****P* < 0.001, and *****P* < 0.0001; n.s., not significant.

**Figure S52.** Flow cytometry gating strategy and representative flow cytometry zebra pot of M1-like (CD80^+^) and M2-like (CD206^+^) TAMs in TME of B16-F10 tumor-bearing mice treated with Saline, PEAMP, P-P_50_, Free Rg, P@Rg, or P@Rg-P_50_.

**Figure S53.** Flow cytometry gating strategy and representative flow cytometry zebra pot of CD8a^+^ and CD4^+^ T cells in TME of B16-F10 tumor-bearing mice treated with Saline, PEAMP, P-P_50_, Free Rg, P@Rg, or P@Rg-P_50_.

**Figure S54.** Flow cytometry gating strategy and representative flow cytometry zebra pot of CD8a^+^ and CD4^+^ T cells in spleen of B16-F10 tumor-bearing mice treated with Saline, PEAMP, P-P_50_, Free Rg, P@Rg, or P@Rg-P_50_.

**Figure S55.** Flow cytometry gating strategy and representative flow cytometry zebra pot of MDSCs in TME of B16-F10 tumor-bearing mice treated with Saline, PEAMP, P-P_50_, Free Rg, P@Rg, or P@Rg-P_50_.

**Figure S56.** Flow cytometry gating strategy and representative flow cytometry zebra pot of Tregs in TME of B16-F10 tumor-bearing mice treated with Saline, PEAMP, P-P_50_, Free Rg, P@Rg, or P@Rg-P_50_.

**S3 Supplementary Tables**

**Table S1.** Polymerization of Chol-PItEG_m_.

| Copolymers | Feed ratios of initiator to monomer | Initiator | Monomer |
| --- | --- | --- | --- |
| Chol-PItEG_20_ | 1:20 | 0.012 mmol | 0.25 mmol |
| Chol-PItEG_30_ | 1:30 | 0.012 mmol | 0.37 mmol |
| Chol-PItEG_50_ | 1:50 | 0.012 mmol | 0.62 mmol |

**Table S2.** Polymerization results of Chol-PItEG_m_.

| Polymer^a^ | *M*_n_ | PDI^b^ | DP^c^ |
| --- | --- | --- | --- |
| Chol-PItEG_20_ | 8738 | 1.07 | 24 |
| Chol-PItEG_30_ | 11855 | 1.17 | 32 |
| Chol-PItEG_50_ | 19527 | 1.13 | 55 |

^a^The Chol-PItEG_m_ was synthesized according to Supplementary Table 1.

^b^Polydispersity index.

^c^Degree of polymerization.

**Table S3.** Size distribution and zeta potential of P@Rg-P_m_ measured by DLS.

| Sample | Size (nm) | Zeta potential (mV) |
| --- | --- | --- |
| P@Rg-P_20_ | 183.8 ± 15.3 | 17.1 ± 2.2 |
| P@Rg-P_30_ | 185.7 ± 8.4 | 16.9 ± 3.5 |
| P@Rg-P_50_ | 190.0 ± 5.2 | 17.8 ± 0.7 |

**Table S4.** Polymerization of HO-PItEG_m_.

| Copolymers | Feed ratios of initiator to monomer | Initiator | Monomer |
| --- | --- | --- | --- |
| HO-PItEG_20_ | 1:20 | 10.0 mg, 0.022 mmol | 150.2 mg, 0.445 mmol |
| HO-PItEG_30_ | 1:30 | 10.0 mg, 0.022 mmol | 225.3 mg, 0.668 mmol |
| HO-PItEG_50_ | 1:50 | 10.0 mg, 0.022 mmol | 375.4 mg, 1.113 mmol |

**Table S5.** Polymerization results of HO-PItEG_m_.

| Polymer^a^ | Solvent | *M*_n_ | PDI^b^ | DP^c^ |
| --- | --- | --- | --- | --- |
| HO-PItEG_20_ | THF | 8806 | 1.16 | 25 |
| HO-PItEG_30_ | THF | 11875 | 1.17 | 34 |
| HO-PItEG_50_ | THF | 19089 | 1.12 | 55 |

^a^The HO-PItEG_m_ was synthesized according to Supplementary Table 4.

^b^Polydispersity index.

^c^Degree of polymerization.

**Table S6.** List of full names and abbreviations of nano-agents.

| PEAMP | Blank PEAMP nanovesicles |
| --- | --- |
| P@R | PEAMP nanovesicles loaded with R848 |
| P@g | PEAMP nanovesicles loaded with gp100 peptides |
| P@Rg | PEAMP nanovesicles loaded with R848 and gp100 peptides |
| P@Rg-P_20_ | P@Rg nanovesicles decorated with helical chains Chol-PItEG_20_ |
| P@Rg-P_30_ | P@Rg nanovesicles decorated with helical chains Chol-PItEG_30_ |
| P@Rg-P_50_ | P@Rg nanovesicles decorated with helical chains Chol-PItEG_50_ |
| P-P_20_ | PEAMP nanovesicles decorated with helical chains Chol-PItEG_20_ |
| P-P_30_ | PEAMP nanovesicles decorated with helical chains Chol-PItEG_30_ |
| P-P_50_ | PEAMP nanovesicles decorated with helical chains Chol-PItEG_50_ |

**Table S7.** Characteristics of primers.

| Gene | | Primers |
| --- | --- | --- |
| TNF-α | Forward（5’ to 3’） | GATCGGTCCCCAAAGGGATG |
|  | Reverse（5’ to 3’） | CCACTTGGTGGTTTGTGAGTG |
| IL-1β | Forward（5’ to 3’） | TCGCAGCAGCACATCAACAAGAG |
|  | Reverse（5’ to 3’） | TGCTCATGTCCTCATCCTGGAAGG |
| IL-12 | Forward（5’ to 3’） | TGGTTTGCCATCGTTTTGCTG |
|  | Reverse（5’ to 3’） | ACAGGTGAGGTTCACTGTTTCT |
| iNOS | Forward（5’ to 3’） | TGCTTTGTGCGAAGTGTCAG |
|  | Reverse（5’ to 3’） | CCCTTTGTGCTGGGAGTCAT |
| YAP1 | Forward（5’ to 3’） | GGCTCTAAAGAACCCGAACC |
|  | Reverse（5’ to 3’） | GCAGCTGAAGAAACCACCTC |
| Piezo1 | Forward（5’ to 3’） | TACGCCGAGGTGTGCTGGAC |
|  | Reverse（5’ to 3’） | GCTGGTGTCGTCTGTCATGCTAC |
| β-actin | Forward（5’ to 3’） | GTGCTATGTTGCTCTAGACTTCG |
|  | Reverse（5’ to 3’） | ATGCCACAGGATTCCATACC |

**Table S8.** Characteristics of antibodies.

| Antibodies | Clone/catalog number | Fluorescence-labeled | Application | Brand |
| --- | --- | --- | --- | --- |
| Anti- mouse-CD80 | 16-10A1 | PE | Flow | eBioscience |
| Anti-mouse-CD86 | GL1 | FITC | Flow | eBioscience |
| Anti-mouse-MHC II | 21-1A6 | FITC | Flow | eBioscience |
| Anti-mouse-CD206 | MR6F3 | APC | Flow | eBioscience |
| Anti-mouse-F4/80 | BM8 | PE-Cy7 | Flow | eBioscience |
| Anti-mouse-F4/80 | BM8 | AF700 | Flow | eBioscience |
| Anti-mouse-CD45 | 30-F11 | PE-Cy7 | Flow | eBioscience |
| Anti-mouse-CD3 | 17A2 | PE | Flow | eBioscience |
| Anti-mouse-CD3 | 17A2 | APC | Flow | eBioscience |
| Anti-mouse-CD4 | GK1.5 | FITC | Flow | eBioscience |
| Anti-mouse-CD8a | 53-6.7 | APC | Flow | Biolegend |
| Anti-mouse-CD8a | 53-6.7 | PE | Flow | eBioscience |
| Anti-mouse-CD11b | M1/70 | FITC | Flow | eBioscience |
| Anti-mouse-Gr-1 | RB6-8C5 | APC | Flow | Biolegend |
| Anti-mouse-CD25 | 3C7 | APC | Flow | Biolegend |
| Anti-mouse-FOXP3 | MF-14 | PE | Flow | Biolegend |
| Anti-mouse-CD44 | MA1-10229 | FITC | Flow | eBioscience |
| Anti-mouse-CD44 | MA1-10229 | AF700 | Flow | eBioscience |
| Anti-mouse-CD62L | IM7 | AF700 | Flow | eBioscience |
| Anti-mouse-CD127 | A7R34 | FITC | Flow | eBioscience |
| Alexa Fluor® 647 Anti-active YAP1 | AB225440 | / | IF | Abcam |
| Rabbit-to-mouse-anti-YAP1 | AB205270 | / | WB | Abcam |
| Rabbit-to-mouse-anti- YAP1 (phospho S127) | AB76252 | / | WB | Abcam |
| Rabbit-to-mouse-anti-NF-κB p65 | HA721307 | / | WB | HUABIO |
| Anti-mouse-anti-GAPDH | EM1101 | / | WB | HUABIO |

**S4 Supplementary movie legends**

**Movie S1-**Representative video of force measurements during five cycles of mPEG_2000_-beads/P_m_-beads approaching macrophages under brightfield imaging via a single-molecule optical tweezer system.

**Movie S2-**Representative video of Fluo-4 fluorescence intensity changes in macrophages treated with PBS for 10 min, with or without GsMTx4.

**Movie S3-**Representative video of Fluo-4 fluorescence intensity changes in macrophages treated with PEAMP for 10 min, with or without GsMTx4.

**Movie S4-**Representative video of Fluo-4 fluorescence intensity changes in macrophages treated with P-P_20_ for 10 min, with or without GsMTx4.

**Movie S5-**Representative video of Fluo-4 fluorescence intensity changes in macrophages treated with P-P_30_ for 10 min, with or without GsMTx4.

**Movie S6-**Representative video of Fluo-4 fluorescence intensity changes in macrophages treated with P-P_50_ for 10 min, with or without GsMTx4.

**Movie S7-**Representative video of Fluo-4 fluorescence intensity changes in macrophages treated with Yoda1 for 10 min, with or without GsMTx4.
